# Empowering personalized oncology: evolution of digital support and visualization tools for molecular tumor boards

**DOI:** 10.1186/s12911-024-02821-8

**Published:** 2025-01-16

**Authors:** Cosima Strantz, Dominik Böhm, Thomas Ganslandt, Melanie Börries, Patrick Metzger, Thomas Pauli, Andreas Blaumeiser, Alexander Scheiter, Ian-Christopher Jung, Jan Christoph, Iryna Manuilova, Konstantin Strauch, Arsenij Ustjanzew, Niklas Reimer, Hauke Busch, Philipp Unberath

**Affiliations:** 1 Medical Informatics, Friedrich-Alexander-Universität Erlangen-Nürnberg, Erlangen, Germany; 2 Medical Center for Information and Communication Technology, Universitätsklinikum Erlangen, Friedrich-Alexander-Universität Erlangen-Nürnberg, Erlangen, Germany; 3 Institute of Medical Bioinformatics and Systems Medicine, Medical Center-University of Freiburg, Faculty of Medicine, University of Freiburg, Freiburg, Germany; 4 German Cancer Consortium (DKTK), Partner site Freiburg, a partnership between DKFZ and Medical Center-University of Freiburg, Freiburg, Germany; 5 Institute of Pathology, University of Regensburg, Regensburg, Germany; 6 Bavarian Center for Cancer Research / BZKF, Regensburg, Germany; 7 Faculty of Medicine and University Hospital Carl Gustav Carus, TUD Dresden University of Technology, Dresden, Germany; 8 Junior Research Group (Bio-)medical Data Science, Faculty of Medicine, Martin-Luther-University Halle-Wittenberg, Halle, Germany; 9 Institute of Medical Biostatistics, Epidemiology and Informatics (IMBEI), University Medical Center of the Johannes Gutenberg-University Mainz, Johannes Gutenberg-University Mainz, Mainz, Germany; 10 Group for Medical Systems Biology, Lübeck Institute of Experimental Dermatology, Universität zu Lübeck, Lübeck, Germany; 11 University Cancer Center Schleswig-Holstein, Lübeck, Germany; 12 Medical Data Integration Center, University Hospital Schleswig-Holstein, Lübeck, Germany; 13 SRH University of Applied Sciences, Fürth, Germany; 14 Bavarian Cancer Research Center (BZKF), Erlangen, Germany

**Keywords:** Molecular tumor board, Personalized oncology, Precision medicine, Genomics, Annotation, Clinical decision support systems, Visualization, cBioPortal, Requirements analysis, User-centered design

## Abstract

**Background:**

Molecular tumor boards (MTBs) play a pivotal role in personalized oncology, leveraging complex data sets to tailor therapy for cancer patients. The integration of digital support and visualization tools is essential in this rapidly evolving field facing fast-growing data and changing clinical processes. This study addresses the gap in understanding the evolution of software and visualization needs within MTBs and evaluates the current state of digital support. Alignment between user requirements and software development is crucial to avoid waste of resources and maintain trust.

**Methods:**

In two consecutive nationwide medical informatics projects in Germany, surveys and expert interviews were conducted as stage 1 (*n* = 14), stage 2 (*n* = 30), and stage 3 (*n* = 9). Surveys, via the SoSci Survey tool, covered participants' roles, working methods, and support needs. The second survey additionally addressed requirements for visualization solutions in molecular tumor boards. These aimed to understand diverse requirements for preparation, implementation, and documentation. Nine semi-structured expert interviews complemented quantitative findings through open discussion.

**Results:**

Using quantitative and qualitative analyses, we show that existing digital tools may improve therapy recommendations and streamline MTB case preparation, while continuous training and system improvements are needed.

**Conclusions:**

Our study contributes to the field by highlighting the importance of developing user-centric, customizable software solutions that can adapt to the fast-paced environment of MTBs to advance personalized oncology. In doing so, it lays the foundation for further advances in personalized medicine in oncology and points to a shift towards more efficient, technology-driven clinical decision-making processes. This research not only enriches our understanding of the integration of digital tools into MTBs, but also signals a broader shift towards technological innovation in healthcare.

**Supplementary Information:**

The online version contains supplementary material available at 10.1186/s12911-024-02821-8.

## Background

Molecular tumor boards (MTBs) are multidisciplinary committees of experts from (molecular) pathology, hematology/oncology, human genetics, bioinformatics, molecular biology, and additional clinical disciplines (e.g., radiology, radiotherapy, nuclear medicine). They represent a cornerstone of personalized medicine by guiding the treatment process for patients with exhausted standard therapies based on the integration of multiomics diagnostics [1, 2], combining state-of-the-art next-generation sequencing (NGS) methods [3], embedded in the German Network for Personalized Medicine (DNPM)^1^ [4].

MTBs aim to enhance patient care and drive research by integrating clinical and molecular data. Through NGS and expert interpretation, data are transformed into visual presentations that inform advanced molecular diagnostics. This approach yields personalized therapy recommendations and generates novel research insights and potential multiomics-based therapeutic indications [4–6].

Multiple German university hospitals now have implemented an MTB, and non-academic hospitals are following this trend. Due to their pioneering role and interface function between different disciplines and between research and clinical practice, MTBs pose significant challenges [3, 7–10]. Clinicians are faced with increasingly complex genetic information, a growing number of available therapies and published clinical trials offering a combination of different molecular targeted (and non-targeted) therapeutic options to choose from. Further issues concern (i) structuring the documentation of molecular data next to clinical data, (ii) harmonizing treatment recommendations beyond clinical guidelines in accordance with internal clinical and patient protection guidelines [7], (iii) harmonizing local implementations of interfaces to patient records and laboratory systems. Although the transition from paper-based solutions to structured tools such as the electronic health record (EHR) is well advanced, patient-specific MTB therapy recommendations remain documented as unstructured free-text fields.

Furthermore, MTBs often vary widely in terms of composition, tasks, and workflows, which inevitably leads to variation in the quality of oncology care [3, 5, 7, 8, 11, 12]. The clinical interpretation of molecular data is typically the bottleneck of the MTB process, as the physician must gather the relevant information from various sources. Due to this complex, often manual, and time-consuming process, the number of patients who can benefit from personalized therapy is substantially low [13, 14]. This results in proprietary software solutions, free text without coordinated data structure, and insufficient traceability of treatment evidence and decisions, all of which hamper retrospective or follow-up studies, data exchange, and research projects [12, 15]. Therefore, it is of utmost importance to document MTB recommendations and decisions based on molecular diagnostics in a structured and digital way to standardize treatment recommendations and medical outcomes across clinics and sites with uniform data formats and reporting rules, with the aim of improving patient care, as Buechner et al. state [7, 16]. To address this issue, the MIRACUM (Medical Informatics in Research and Care in University Medicine) consortium initiated Use Case 3 (UC3) in 2018 as one of the use cases of the Medical Informatics Initiative (MII), which aims to provide IT and bioinformatics support for the translation and visualization of data analyzed in MTBs [17].

The cBio Cancer Genomics Portal (cBioPortal) was selected as a suitable starting platform to visualize the data generated from NGS analysis, e.g., through the MIRACUM-Pipeline [18]. It offers tools for exploring, visualizing, and analyzing large-scale cancer genomic data. This toolkit supports case preparation, discussion, documentation, visualization, and communication of treatment recommendations in MTBs and is suitable to replace current university hospital practices [15, 19–21]. The platform cBioPortal was adapted and integrated into hospital information systems (HIS) for data exchange with electronic patient records and laboratory systems, aiming to serve as a future Clinical Decision Support System (CDSS) [7, 20, 22].

According to Sim et al., a CDSS is defined as ‘software designed to be a direct aid to clinical decision-making, in which the characteristics of an individual patient are matched to a computerized clinical knowledge base and patient-specific assessments or recommendations are then presented to the clinician or the patient for a decision’ [23]. Research shows that CDSSs have the potential to improve and accelerate the diagnosis, but knowledge regarding the utilization of CDSSs for diagnosing acute and rare diseases in primary care settings is limited [24]. Studies have identified several limitations and challenges to CDSSs and their use, such as poor workflow integration and a lack of acceptance or trust [25]. Overcoming these barriers requires a user-centered design (UCD) and integration with existing hospital systems for seamless data exchange among electronic patient files, laboratory systems, and the extended MTB-cBioPortal [26, 27]. For this, PM4Onco ("Personalized Medicine for Oncology") was set up in 2023 in the expansion phase of the MII as a joint project of all four consortia of the MII,^2^ funded by the National Decade Against Cancer of the German Federal Ministry of Education and Research [28].^3^

As part of PM4Onco, this study examines how visualization optimizes complex data capture in MTBs, structuring information to ease cognitive load in decision-making. It is unclear if current visualization tools meet clinicians' needs. We therefore surveyed users to inform cBioPortal’s further development [27, 29].

MTB case interpretations involving the evaluation of multiple biomarkers and the recom-mendation of a therapy combination and trial participation is only possible in the synopsis of all available information. Full automation without human expertise in molecular pathology or oncology is infeasible, aligning with integrative platforms like cBioPortal [1, 30].

In considering this, we hypothesize that data visualization could be a strategy to reduce the time required to preparing MTB cases. We aim to show the progression of software and more specifically visualization requirements for MTBs as well as the current state of support provided through previous initiatives. Our findings are intended to enable further user-centered development of software support and visualization tools for MTBs.

Our study revealed how the implemented innovations can reduce MTB preparation time, may improve therapy suggestions, and provide better support than existing applications.

## Methods

**Fig. 1:**
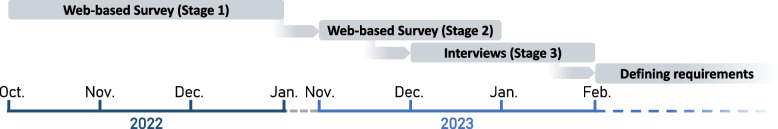
Design of iterative requirements analysis

To generate starting points for further development of an MTB visualization platform, we conducted quantitative and qualitative analyzes through two surveys and a series of expert interviews, as part of two consecutive nationwide projects in German medical informatics; surveys were administered during the final phase of the project MIRACUM-UC3 stage 1 (with *n* = 14 participants in the quantitative study) and at the outset of the subsequent project PM4Onco, stage 2 (*n* = 30) and stage 3 (*n* = 9). These surveys were conducted anonymously using the SoSci Survey platform [31] and addressed three main areas as shown in Table 1. All questionnaires had 3 parts (A, B and C), which contained different questions depending on the stage (1, 2 & 3), for details see Additional Files 1, 2, 3, and 4. The goal was to gather a detailed understanding of the diverse needs associated with the preparation, implementation, and documentation of MTBs. This data was further enriched by a series of semi-structured expert interviews (involving *n* = 9 interviewees) designed to deepen the quantitative findings with insightful discussions. Therefore, we developed an interview guide to structure the expert interviews conducted in stage 3 (see Additional File 5). An overview of the stepwise analysis process is shown in Fig. 1. Table 2 provides an outline of the design and participants across all stages.

**Table 1:** Survey structure and approach for assessing MTB platform adoption and requirements: a multistage inquiry

Part	Stage	Questions
A	1	Questions about the conventional way of working without using the MTB platform
	2	Questions about the current way of working
B	1 B1	Questions on the use of the MTB platform
	1 B2	Questions about the current way of working with the MTB platform
	1 B3	Questions about acceptance of the MTB platform
	2	Specific requirements for visualization tools in MTBs
C	1 & 2	Socio-demographic questions and activity in the MTB

**Table 2:** Design and participants

**Stage**	*Methodology*	*Focus*	*Time Frame*	*Target Group*	*Common Aspects*
1	Summative Evaluation, Online Survey	Assessing process quality (e.g., procedures related to patient care) and outcome quality (e.g., user satisfaction, improvement of therapy recommendations) and data on traditional workflows, user experiences, and system acceptance.	Between October and December 2022	Tumor board preparation teams (*n *= 4) and Specialist teams (*n *= 10) from six German university hospitals	• Retrospective, open, uncontrolled cross-sectional study, questions were group-specific and included multiple-choice, rating scales, and free text formats, with a ‘not applicable/can’t say’ option. • No inclusion or exclusion criteria • No financial compensation • Participants provided their consent at the beginning of the survey; those who did not consent were notable to continue. • We followed the theoretical framework according to Donabedian^a^.
2	Follow-up Online Survey	Current workflows and new visualization methods requirements.	Between October and December 2023, with a smaller survey conducted in February 2024 post-cBioPortal release v6.0.0^b^	25 PM4Onco sites across Germany, researchers, and clinical staff	
3	Semi-structured Online Expert Interviews^c,d^	Identifying further specific requirements and feedback essential for the development of effective visualization solutions for MTBs.	Between December 2023 and January 2024	Researchers, and clinical staff from six PM4Onco sites	

^a^Donabedian A. Evaluating the quality of medical care. 1966. Milbank Q. 2005;83(4):691–729

^b^We did not query the location where respondents could be identified, as otherwise, the risk of re-identification would be too high due to the relatively small number of experts in total

^c^We considered the work of Baxter et al. [32] when designing questions targeting (1) status quo in MTB workflow and process, (2) existing technological support, (3) challenges and need for support regarding in MTB workflow and process, (4) perceived quality and problems to the same, (5) requirements on visualization methods, (6) integration, (7) and outlook for visionary features. The interviews lasted 45 minutes on average

^d^Understanding Your Users [Internet]. Elsevier; 2005 [cited 2024 Feb 27]. Available from: https://linkinghub.elsevier.com/retrieve/pii/B9781558609358X50295

Our study focuses on requirements engineering for specific software tools. Requirements analysis, as per IEEE^4^ based on Herrmann [33], involves studying and refining system, hardware, or software requirements. This process includes activities like requirements identification, analysis, specification, and evaluation, which are often conducted iteratively. Our requirements analysis for cBioPortal follows a user-centered development process, integrating iterative prototyping, user feedback, and usability evaluations [34].

All participant responses were captured anonymously. Stage 1 analysis was performed using SPSS^5^ and data analysis was primarily descriptive (frequencies, means, medians, interquartile ranges, and standard deviations). Stage 2 analysis was conducted in Python (version 3.12.1). For questions requiring a rating scale from 1 to 5, the plot-likert^6^:0.5.0 library was utilized for visualization, while multiple-choice questions were visualized using the ydata-profiling [35] :4.7.0 and pandas [36] :2.1.4 libraries.

We anonymized all data from recorded interviews. We then applied a content analysis approach, as suggested by Mayring and Kuckartz & Rädiker, respectively [32, 37, 38]. We started with the initial text work: marking important text passages, writing memos (MAXQDA: memos for ideas [38]), then we developed thematic main categories, deductively derived from the questions of the interview guide. We coded the entire material with the main categories (rough coding), which led to ‘sentence’ as the smallest coding. The coded system had to be understandable taken alone and finally to the compilation of all text passages coded with the same main categories. We then inductively determined subcategories from the material. The final step involved coding the complete material with the differentiated category system. We performed and reported the interviews in accordance with the Consolidated Criteria for Reporting Qualitative Research (COREQ) [39].

Three authors (C.S., P.U., and D.B.) read and independently coded each of three randomly selected interviews into codebooks. In this process, codes were assigned for the respective answers to the questions. If an interviewee gave a very similar answer to a question, the same code was used. Afterward, the authors compared the independently created codes and merged them into a single codebook. Owing to a high coding agreement of 85%, the authors then proceeded to code the remaining six interviews independently (three each). In the case of incomplete interviews, only the answered questions were considered for coding.

The personal characteristics (names) of all interview participants were replaced by codes (pseudonymized) as follows:

- Interviewer = I
- Assessor 1 to 3 = BE1, …, BE3
- Interviewee 1 to 9 = BF1, …, BF9

## Results

Based on the survey results we obtained a clearer picture of the current state of digital support and visualization tools for MTBs across a variety of German hospitals. The surveys revealed that previous development may improve the quality of therapy suggestions in terms of reducing the efforts for preparing molecular tumor board cases. Respondents tend to prefer the digitally supported process over the conventional one. However, the evaluation indicated a need for continuous training, as well as an expansion of the functionality and an improvement of the presentation of results in supporting systems. These findings together with the requirements extracted from the expert interviews form a strong basis for improving and developing current and new tools and visualization solutions specifically tailored to the needs of MTBs. The results reflect the need to develop software with an emphasis on usability and customizability.

### cBioPortal

The open-source tool cBioPortal is a web-based platform for exploring and visualizing multidimensional cancer genomics data, hosted by the Memorial Sloan Kettering Cancer Center (MSKCC). This public instance enables researchers to access a collection of curated cancer studies and perform detailed analyses. Additionally, cBioPortal supports the setup of local instances, allowing institutions to integrate the platform into their in-house IT infrastructure for secure analysis of sensitive data while complying with data protection regulations [5, 40].

For MTBs, the customized MTB-cBioPortal extends the original platform with features tailored to MTB workflows. Key enhancements include two new tabs: (1) an automated search for clinical trials based on genomic and clinical data, integrating ClinicalTrials.gov for trial matching [20], and (2) a structured and standardized documentation feature for MTB treatment recommendations [41]. Additional customizations streamline integration with hospital information systems (HIS), such as linking therapy recommendations to order IDs for electronic health record compatibility and adopting FHIR-based standards to improve data interoperability.

**Fig. 2:**
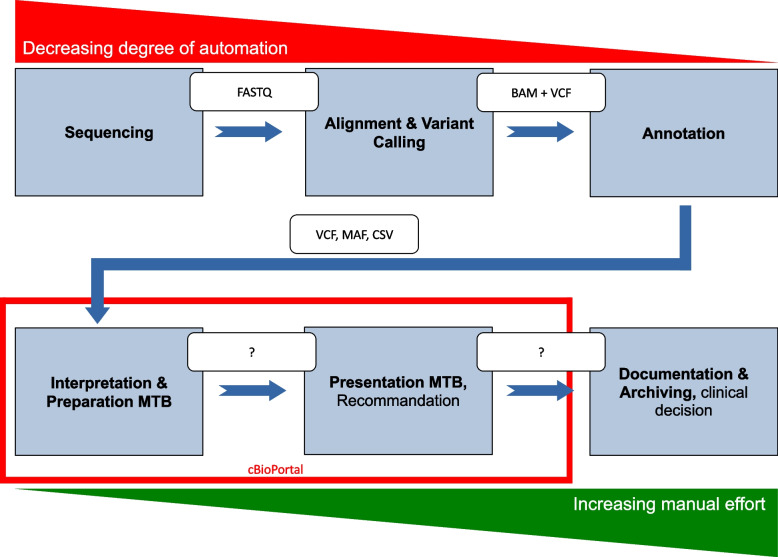
MTB process model. A molecular analysis is ordered when a patient is admitted to the MTB. Lab technicians sequence the tumor to produce the raw sequencing data in FASTQ format (https://compgenomr.github.io/book/fasta-and-fastq-formats.html) (Sequencing). Sequencing data is aligned to a reference genome, and differences between the sequenced genome and the reference genome and variants (mutations) specific to the tumor are identified (Alignment & Variant Calling). The alignment process on the reference genome generates a BAM file (Binary Alignment Map) and a corresponding BAI file (Binary Alignment Index). Variant calling tools generate files in the Variant Call Format (VCF). Mutation Annotation Format (MAF) is a tab-delimited text file with aggregated mutation information from VCF files and is generated on a project level. From this point on, cBioPortal is deployed. The research expert then creates a case in the MTB tool by bringing together all relevant case data from different sources. Variants are annotated to determine their potential impact on protein function and relevance to cancer biology (Annotation). The annotated variants are interpreted in the context of existing scientific knowledge, clinical guidelines, and patient-specific factors. The findings are summarized for presentation at the Molecular Tumor Board meeting (Interpretation & Preparation for MTB). Variants are categorized based on their therapeutic relevance according to external sources. The research expert selects the most relevant variants and assigns them to the therapies identified in the literature review. The recommendations and rationale behind the proposed treatment plan are documented for the patient's medical records. Additionally, the sequencing data and MTB deliberations are archived for future reference and research purposes (Documentation & Archiving)

**Fig. 3:**
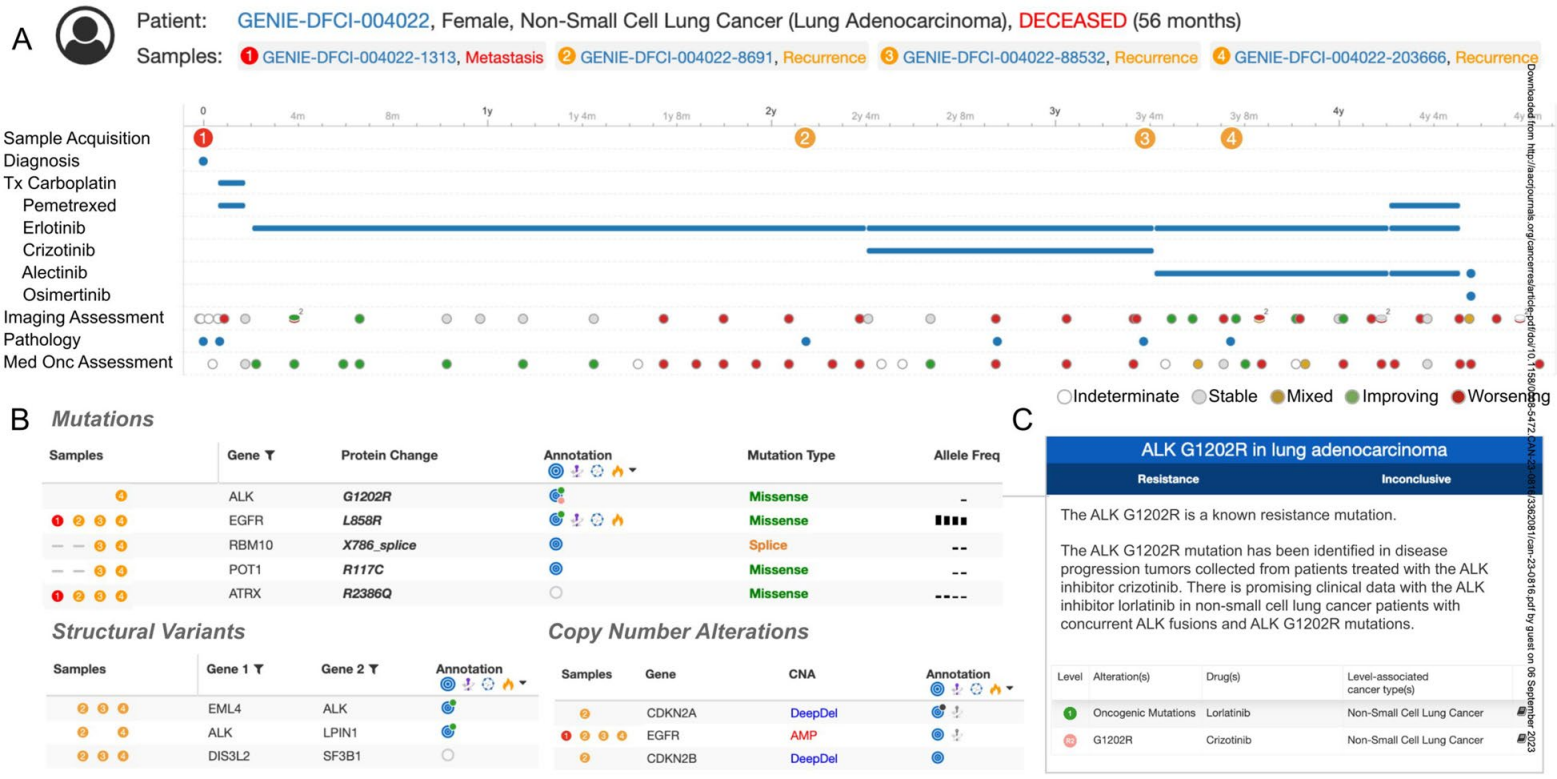
Visualizing longitudinal clinical and genomic profiles of a patient in GENIE BPC A. The timeline provides a comprehensive overview of biopsies, resections, diagnoses, treatment regimens, and diagnostic assessments from medical imaging and oncology. B. Detailed genomic event tables offer insights into mutations, structural variants, and copy-number alterations observed in each sample. Specifically, the mutations table includes information on protein effects and variant allele frequencies. The 'samples' column specifies the sample(s) where each event was detected, with a dash indicating non-profiling for a particular gene. Genomic events are annotated with data from external resources, such as OncoKB, represented by a blue target icon. Hovering over these icons provides users with additional information via tooltips. C. An example tooltip demonstrates the OncoKB annotation for ALK G1202R, indicating that this mutation detected in sample 4 is a known resistance mutation against crizotinib. Example data adopted from the public cBioPortal (https://www.cbioportal.org, https://genie.cbioportal.org)

Figure 2 indicates how cBioPortal is integrated into the MTB process. Figure 3 shows the features currently implemented in cBioPortal and possible starting points for new visualization techniques.

**Fig. 4:**
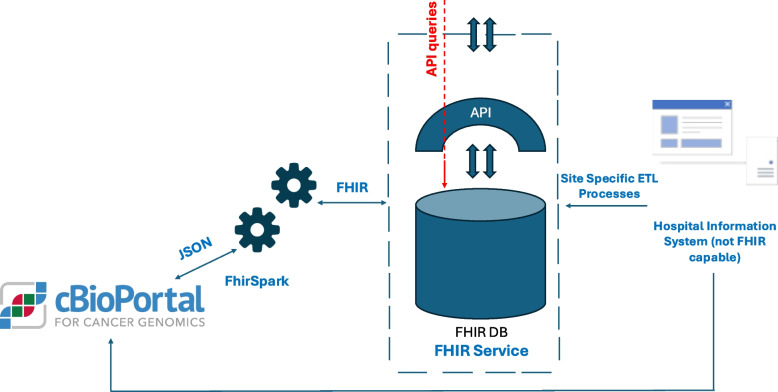
(in reference to Fig. 2 of Reimer et al. [42]): Proposal for integrating cBioPortal into the clinical infrastructure. Site-specific ETL processes are used to get the data from HIS in FHIR. FhirSpark serves as a mediation layer between cBioPortal and a FHIR-capable server. Rather than relying on a legacy database that lacks FHIR, which would require synchronization or mapping to FHIR, instead FHIR is implemented as the native storage format. In addition, there is the initial, direct way to get data into cBioPortal

CBioPortal’s flexibility allows it to support both research and clinical applications. However, its integration into clinical environments often requires further local adaptations, such as custom workflows, preprocessing pipelines, and compatibility with standardized data formats like Mutation Annotation Format (MAF) and harmonized FHIR profiles. Figure 4 illustrates a generic integration of cBioPortal into the clinical infrastructure, ensuring interoperability by leveraging FHIR standards. Further technical details, including deployment processes, system architecture, and data management, are available in the appendix (Additional File 10). For in-depth information on cBioPortal’s capabilities and its extensions for MTB use cases, see [de Bruijn et al., Gao et al., Cerami et al.] and [Buechner et al., Reimer et al., Renner et al.].

### Quantitative insights from survey results

Table 3 summarizes the details and characteristics of the participants from all three phases, whose contributions provided the basis for identifying the challenges associated with using the extended cBioPortal and/or PDF report,^7^ such as manual data input due to lacking user interfaces, insufficient databases for therapy decision support, and collaboration obstacles."

**Table 3:** Details about participants

**Stage**	*Number of Participants*	*Demographics of Participants*	*Disciplines/Position (Number)*	*Details MTB (Participation)*
1	Four Participants (two female, one male, one unspecified) - Tumor board preparation teams	Respondents were on average 40 years old (range 33 to 45 years)	Hematology/Oncology (2), Bioinformatics (1), Systems Medicine and Bioinformatics (1)	• They had been members of the MTB for three years on average. • The MTB took place on average four times per month with an average of four cases per session.
	10 Participants (four female, six male) - Specialist teams	Respondents were on average 40 years old (range 28 to 45 years)	Hematology/Oncology (9), Bioinformatics (1)	• The majority always attended the MTB sessions. • The MTB took place on average twice per month with an average of 18 cases per session.
2	25 Participants (11 female, seven male, seven not specified)	Respondents were on average 37 years old (range 25 to 50 years)	Hematology/Oncology (11), Molecular Biology (4), Human Genetics (2), Gynecology (2), Systems Medicine and Bioinformatics (2), Neurosurgery (1), and not specified (7)	• They had been members of the MTB for an average duration of three years. • The MTB took place on average four times per month with an average of nine cases per session.
3	Nine Participants (seven female, two male)	was not queried	Hematologist/Oncologist (5), Coordinator (2), Resident (1), Neuroscientist (1).	was not queried

The extended cBioPortal faces underutilization due to manual data transfer and limited access. Integration of the extended cBioPortal into existing systems is considered rather good and useful, preferred over conventional methods. The PDF report, though useful, faces underutilization due to existing electronic documentation. Overall, respondents perceive improvements in process efficiency, result accuracy, and support for their work with the extended cBioPortal and PDF report. Users reported both a reduction in preparation time and an improved feeling of tool support. For details see Additional File 6.

**Fig. 5:**
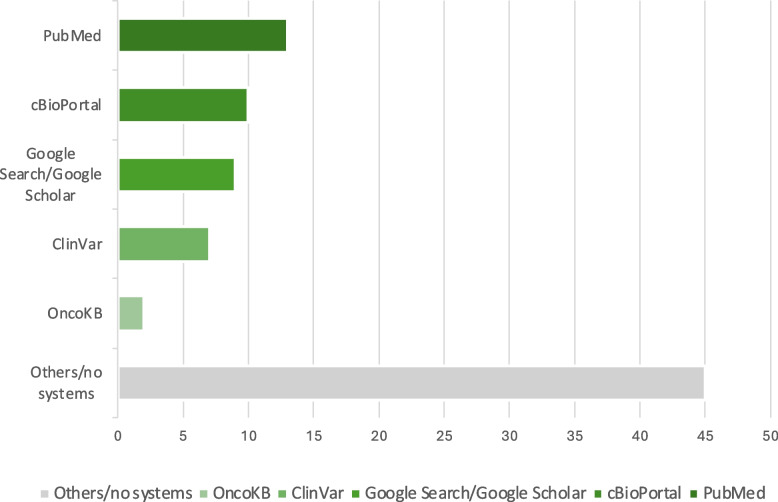
Distribution of applied systems / applications / websites for the preparation of the MTB. (Open question with two possible answers: 1) I do not use any systems / applications / websites for MTB preparation and 2) So far, I have used the following systems / applications / websites for MTB preparation.)

**Fig. 6:**
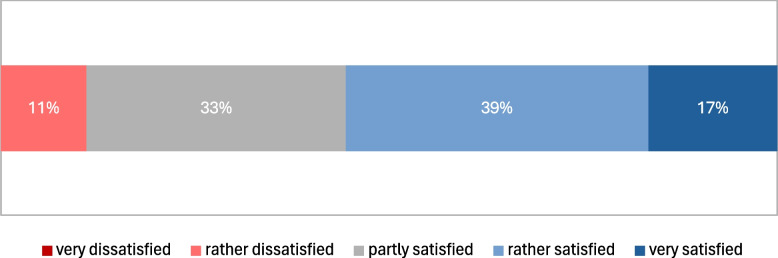
Satisfaction with the clarity of the presentation of the data for the case review for preparing the MTB

The survey data analysis revealed that a variety of sources, including PubMed, cBioPortal, Google Search, and ClinVar,^8^ were utilized for MTB preparation, reflecting the interdisciplinary nature of the process (Fig. 5). Most users had experience with cBioPortal, primarily for MTB preparation and research purposes. Users generally reported satisfaction with traditional methods of preparing for the MTB. However, they identified significant gaps in the current systems, particularly regarding electronic support and the efficiency of search functionalities (Fig. 6).

**Fig. 7:**
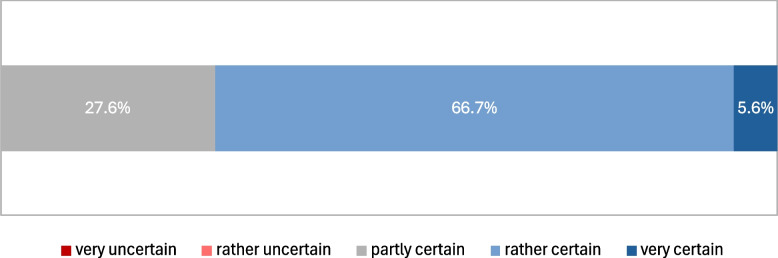
Certainty of being well-informed based on the available data for a case and having made the optimal interpretation

**Fig. 8:**
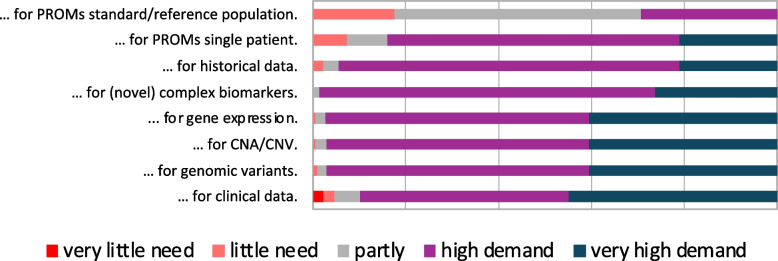
Requirement for novel visualization methods along different data types

Users reported spending a considerable amount of time on data interpretation, particularly for complex cases, highlighting the necessity for more efficient analysis tools. While users generally found the available data to be accurate and complete and found an optimal interpretation for themselves based on this (see Fig. 7), they expressed a need for supportive tools for data analysis due to the complexity of interpretation. Current visualization methods predominantly involve PowerPoint slides/PDF and downstream cBioPortal, indicating a manual approach to data presentation. There is a lack of systems for visualizing Patient-Reported Outcome Measures (PROMs) in the context of MTBs, indicating a need for development in this area. Additionally, there is a high demand for additional visualization methods, particularly for molecular biology data, emphasizing the necessity for specialized and advanced visualization options (Fig. 8).

Fifteen out of 25 respondents consider the customizability of the user interface to be crucial, emphasizing its importance for software efficiency. Key obstacles in MTB preparation include the absence of interfaces and media discontinuities, the challenge of consolidating numerous annotation sources, and the absence of a tailored case presentation format, all of which hinder seamless and efficient preparation processes. For details see Additional File 7.

The small separate survey showed, as in the stage 2, in case of electronically supported preparation for the MTB without using the MTB platform, that the search is characterized by a high expenditure of time (search time ranges from an average of 30 min for simple cases to 90 min for complex cases) due to manual search input and that users tend not to feel supported or only partially supported electronically, also due to the complexity of the data presented. Nevertheless, they rate the data as very complete and accurate and are more confident in the treatment recommendations made on this basis (although at the cost of longer searches). Use of the extended OncoKB, of the trial search / "ClinicalTrials.gov" tab and documentation of the therapy recommendation on the patient page / "Therapy Recommendations" option as already implemented in cBioPortal are stated from occasionally [3] to often/always used [9]. They tend to feel electronically supported when preparing for the MTB by using the MTB platform. In addition, they feel satisfied and (rather) supported with the practice of preparation, the time required is rated as (rather) high, time expenditure (search time ranges from an average of 15 min for simple cases to 40 min for complex cases), so time expenditure tends to reduce due to using the MTB platform.

#### Qualitative findings from expert interviews

Based on the results of the quantitative survey, we conducted semi-structured expert interviews to create a comprehensive list of requirements. Even though the interview was designed as an expert interview in mind, we deviated from the guidelines in some cases when (1) the interviewees brought up new topics on their own and (2) they anticipated topics, or (3) revisited aspects of topics already asked about and introduced new ideas/issues. At the end of each interview, we made sure that we had addressed all the topics in the guidelines. To structure all usable statements, we formed superordinate categories based on the guideline and assigned all statements to the appropriate categories. Within these categories, subcategories were derived from the statements and attributed to them.

#### MTB process, current workflow, challenges, and need for support

We asked respondents to describe how they currently prepare, present, and follow up/document a case for the MTB and what they are currently missing, as well as asking about specific aspects of the current process.

In the interviews, it became clear that the extent of manual effort is very high, clearly demonstrated by the description of the MTB process or the data flow: "between MTB and previously clinic or then out of the MTB and back into the clinic" (I, BF1), representative for all conducted interviews. Even more, there are many media discontinuities (“The big media disruption already takes place in Pathology” (I, BF4) and the information for an MTB case is obtained from several data sources: "I look up everything manually from different databases" (I, BF6). Another key aspect was self-programming: “Home-made brand” (I, BF1), i.e., research results are maintained in specially created lists (e.g., Excel). Presentation of the MTB patient was mainly organized in an unstructured form by "copy&paste into KIS^9^ template"(I, BF4) with “double work due to transfer from A to B, very labor-intensive” (I, BF7) from several documents merging into: screenshots, PowerPoint slides, Word documents mainly as “unformatted text” ((I, BF2) containing PDF documents from pathological finding and interpretation of MTB patient’s data. ‘Automation’ as well as ‘Documentation’ have emerged as (inductively) developed sub-categories.

It serves as starting point for an automated connection of the pathology findings "promptly" (I, BF9) to the cBioPortal and an automatic format template out of the cBioPortal for the MTB case presentation. "Ideally, I would annotate the whole thing in a system and get a overview from there, which I present in an MTB." (I, BF7). In addition, "an automated transfer of the data from pathology is desirable […] that we receive the data promptly […], that never actually works." (I, BF7).

Measured in terms of assignable statements, the subcategory ‘Time spent’ (inductively) was the leading category. One statement, which is representative of many statements in this main category, goes as follows:

"The preparation of the individual patients takes an extremely long time or is sometimes extremely time-consuming because you still have to click through the various databases to conduct literature research in order to find studies that you can refer to, and I mainly use Google Scholar and PubMed." (I, BF9).

This statement includes another derived sub-category, ‘Search for studies’ which is an essential step in therapy recommendation and evidence generation. “We check studies with QuickQueck,^10^ BZKF,^11^ Google, and PubMed and look for case reports.” (I, BF4, BF5). The high expenditure of time is since there is "no incredibly standardized procedure" (I, BF1), but accepted in favor of the patient.

This is the starting point for an automatic query of studies embedded in cBioPortal: "downloads the top 10 Google results in the background […], but it is important to see how up to date the results are" (I, BE3, BF9), and "in the best case they contain congress contributions" (I, BF7) and is thus Requirement 1 (R1) for implementation in the next step of further development of MTB-cBioPortal.

#### Visualization techniques

Here, we asked for improvable visualization methods for different types of data (genomic data, CNVs/CNAs, gene expression, clinical data, PROMs, and historical data).

In this context, it became clear that the interviewees would prefer to have ‘clinical and molecular biological data’ transferred from the respective source systems (‘databases’) to the cBioPortal rather than having to manually search for them and enter them manually into specially created lists, as this saves time and reduces possible transfer errors from copying & pasting.

The participants also pointed out that in a best-case scenario, suggestions for entries would be made automatically during the data entry process, perhaps in the form of an intelligent query, which would help in recognizing what further data and test results should be entered.

To evaluate this data, the participants recommended linking the occurrence of certain diagnoses with their probability. The interviewees prefer the data to be presented "at a glance". Further features considered as important were a filter function (“Filter function by variants, entities, annotations etc.” (I, BF6), a cut-off function (“Cutoff for allele frequency of 30%, for example.” (I, BF2)), and a color code. Important information should be “clickable” (I, BF8) and the underlying information should be visible while clinical and molecular biological data should be transferred automatically.

At the same time, we specifically asked about ‘complex biomarkers’,^12^ and “if the findings provide corresponding content, this is also used" (I, BF3), but “these are not yet presented so clearly in cBioPortal” (I, BF1). Likewise, a filter function was mentioned in this context.

Regarding PROMs, the participants recognized the importance of this data for evaluation ('Super important and super useful. But I think that was a separate project, so independent of the follow-up.' (I, BF4)), but they ranked the urgency of integrating it into cBioPortal rather low." Retrospective evaluation of prior therapy recommendations is considered important, but generally more follow-up information should be acquired from and with the patient. Yet, there are also critical voices: “To be honest, not at all, because I find the topic extremely difficult. How to collect it at all. Well, if you needed a platform to collect PROMs, yes, I think so.” (I, BF8). PROMs are not used for therapy recommendations yet (“In principle, it should also be necessary for decision-making in the future.” (I, BF6)).

Questions about ‘historical data’ were anticipated by some participants. (Pre-)therapies, demographic data, and secondary diagnoses should be presented. Very important is the time axis not starting at 0, but at the year of initial diagnosis or the age of the patient, i.e., the relative presentation at a defined point in time, as mentioned: “If you receive an initial diagnosis in 2013, then nothing happens for seven years, then you have a recurrence and then a lot happens within three years. Then you scale this automatically and it would be much clearer.” (I, BF4). In this context, some participants raised the idea of an alert function: “Yes, I think you could imagine a section in front of the mutations, for example, where you can see them one below the other and then, […], a fire icon pops up or something or the target turns red if it is elevated, […]. So, if it's potentially druggable.” (I, BF1).

This serves as the starting point for changes to the timeline, specifically aimed at displaying absolute dates, and is consequently designated as Requirement 2 (R2) for implementation in the next phase of the further development of MTB-cBioPortal.

#### Integration

We asked participants how they would integrate visualization tools into their workflow. It was suggested that cBioPortal should have the option of 'interaction'—"it would be nice if you could give things back interactively." (I, BF5) and for 'site-specific customization'. Furthermore, external/mobile access from outside the hospital network was mentioned as a desirable feature.

#### Visions

We asked participants for ideas or wishes for future-oriented visualization methods. They mentioned ‘IT support’ of the entire MTB process, as well as a ‘broad data integration’ (“Integration of all relevant data.” (I, BF4)). Furthermore, anonymized therapy recommendations by networking the CCCs, cross-site study search (highlighting open arms of studies), and extractable (out of cBioPortal) as ‘MTB report’, as well as extracting evidence were mentioned. The entire code system is available in the Additional File 8.

### Inferred user requirements for future development

We have extracted a comprehensive list of requirements from the synthesis of the quantitative results of the survey and the qualitative findings from the interviews.

The comprehensive data integration and management in MTB software includes integrating diverse sources like EHRs and molecular test results with secure storage to comply with data privacy regulations. Molecular data analysis entails processing genomic sequencing results and providing effective visualization tools for interpretation. Clinical decision support involves algorithms to interpret findings and recommend personalized treatments, integrating with knowledge bases. Collaborative tools facilitate real-time discussions among team members, with support for virtual meetings. Security measures ensure data privacy and compliance, while usability focuses on intuitive interfaces and customizable workflows. Scalability ensures support for increasing data volumes and participants. Interactive and temporal visualization aids in understanding longitudinal data. Training materials and responsive support services are crucial for user adoption and assistance. Besides generating insights into current preparation workflows of MTB cases, for specific requirements see Additional File 9.

**Fig. 9:**
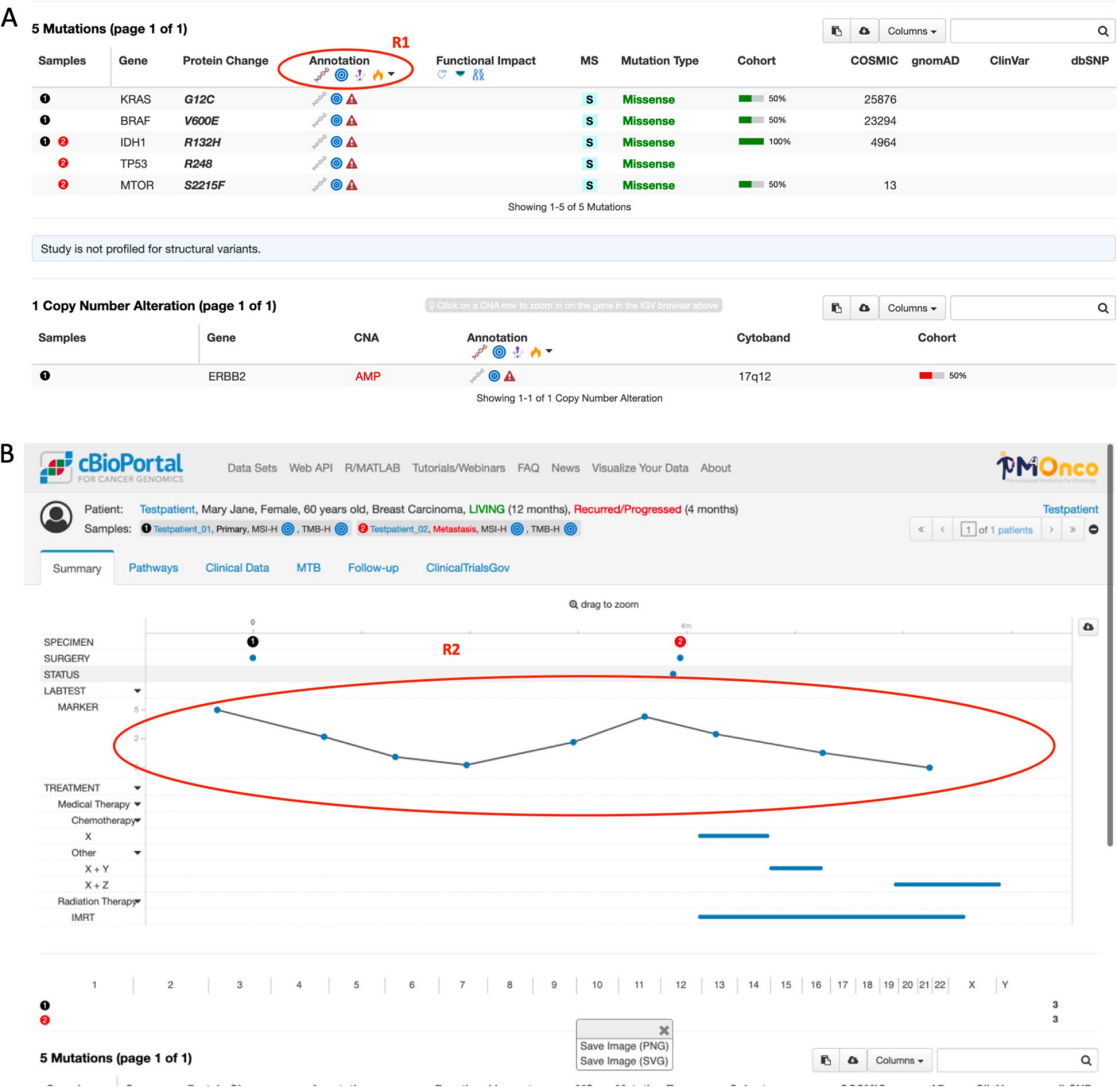
Derived requirements for novel visualization methods R1 and R2. A showing R1: cBioPortal currently supports the annotation of variants with information from several different databases, including OncoKB and CIViC. These databases provide a variety of information including the clinical relevance of mutations, common cancer types, and potential drug targets. We aim to add additional annotations to enhance the preparation process for molecular tumor boards. For example, we intend to include PubMed and Google Scholar annotations that automatically provide search results based on patients’ mutation profiles and clinical data to improve the literature search for potential treatment strategies. B showing R2: We aim to improve cBioPortal’s patient timeline for its application in the preparation and presentation of molecular tumor boards. While the current implementation already provides good visualization of the longitudinal clinical data, we plan to implement changes based on the findings of the requirements analysis, to enhance the readability and improve the integration into clinical processes, e.g. the option to toggle between an absolute and relative date format. We also intend to provide custom visualization solutions for novel data types

Focusing on visualization techniques, we want to highlight two requirements, firstly Interactive Data Visualization (support for interactive charts, graphs, and plots to visualize molecular data and ability to zoom, pan, and filter data – Fig. 9A) and secondly Temporal Visualization (visualization of longitudinal molecular data to track changes in tumor evolution, treatment response, and disease progression over time and time series plots, heatmaps, or animated visualizations to illustrate temporal dynamics of molecular alterations and treatment effects), see Fig. 9B.

## Discussion

In this study we performed a summative usability evaluation based on the results of which new user requirements were derived resulting in a requirements analysis of the extended MTB-cBioPortal and the PDF report. Our requirement analysis was the basic step for further extending cBioPortal with focus on visualization. With the use of surveys and interviews, we determined the user requirements from the oncologists' perspective based on identified user needs. The user requirements dealt with preparing, presentation, and follow up and documentation a case for the MTB. To our knowledge, there is no comparable work to date addressing requirements of software tools for MTBs with a focus on visualization solutions from the perspective of MTB stakeholders.

Limitations of our study are that no randomized controlled trial could be conducted as the most reliable and expected method for verifying the effectiveness of the study conducted [43]; however, co-variables were collected in the survey. For the setting, we had no control over the answering situation (disturbances, distractions, etc.). Regarding implementation, there are possible evaluation effects by the respondents: memory distortions in connection with information about the situation before implementation.

Moreover, the respective surveys are not fully comparable as they have been conducted within different projects and at different points in time. Alternative interview formats such as focus group interviews (1), workshops (2) or design thinking (3) were excluded due to organizational constraints (1 and 2) and the lack of a testable prototype (3).

Studies with small sample sizes are considered critically, which applies to quantitative studies [44], in the present study with stage 1 and the additional survey in stage 2. In qualitative research and in UCD, however, it is inherent to the method that an intensive exchange takes place with a small group of participants with continuous feedback from participants to the design and its development. The results of the qualitative analysis cannot be generalized to larger populations as confidently as quantitative results due to the absence of statistical significance testing.

The samples are not necessarily representative of the status quo in Germany. We could neither include all hospitals (in total, there are 36 university hospitals in Germany)^13^ nor recruit participants from each site involved in this study. The number of participants in the interviews and web-based questionnaires were not evenly distributed across the sites, which adds more power to the responses of those sites. Since procedures can vary between wards as they vary between hospitals, we do not consider this a major limitation. In addition, not all disciplines and their representatives involved in MTBs could be included.

The scope of our results is primarily centered on Germany, specifically within the context of MTBs, and may not be directly applicable to practices in other countries or different fields of application. Given the potential for varying usability issues in different environments, our findings lack generalizability despite cBioPortal's broad international use. Our implementation, part of the MII initiative, is specifically tailored for Germany to harmonize IT system differences across hospitals. The aim is to develop a generic solution and adapting it for other countries might only require minor changes. While similar approaches are being taken by studies from Schapranow et al. and Kahraman et al. [45–47], initiatives like MTPortal connect centers across the EU, in contrast to our national focus [19, 41]. Exploring how other centers or countries address similar challenges remains valuable, supported by our uniformly open-source licensed tools that can serve as a foundation for further development.

The interviews proved to be the most important part of this work as, on the one hand, questions that arose could be clarified immediately and, on the other hand, requirements came to light through intensive discussions that neither the interviewees nor we had previously considered. We were able to identify a total of ten requirements (see Additional File 9), of which six were not yet fully integrated into the cBioPortal at the time of the surveys. Our results support the findings of other studies that the preparation and documentation phases within the MTB process are the key points in terms of software tool support [46, 48].

In addition to molecular characterization of genetic variants, clinical parameters such as the patient's medical history and current general condition are essential for evaluating the possible treatment options, especially considering outcomes of previous treatments. To assess the current tumor status, detailed clinical, pathological, and radiological information from at least the most recent examinations should be provided. In the best-case scenario, this data is provided electronically in real time and easy to navigate (R1). Visualization aspects are desired for the display of (1) time courses (patient history), see R2, the (2) search for similar patients (“patient like me”) and for (3) data types through (1) time point 0 = year, age, and color highlighting of time points, (2) display of approx. ten similar cases and (3) alert function, cut offs, filter functions. Besides those, searching for studies plays an important role and is considered to be (fully) integrated in cBioPortal.

Interviewees mentioned that it was essential to be able to transfer patient data automatically to and from cBioPortal to save time and reduce transfer errors. This requirement is also addressed in related works; for example, Sutton et al. argue that CDSSs can disrupt workflows when used as stand-alone systems and that poor system integration requiring manual data entry is a major barrier to the adoption of diagnostic decision support systems [26]. Nurek et al. consider double data entry as an obstacle to the use of CDSSs [49].

Each MTB session leads to new findings that need to be stored and linked for later reference. Most importantly, the tumor board recommendation for each case discussed must be documented, signed, and returned to the HIS. The tumor board recommendation contains all relevant clinical, pathological, radiological, and molecular information, but also relevant clinical studies, literature, and information on similar cases that were decisive for the recommendation. The idealization here lies in an automatic format template in cBioPortal for case presentation, which can be filled in to replace manually compiled presentations in the MTB in the long term. The presentation can be delivered in a textual format without the need to be visually elaborate if in turn it can be understood rapidly. This approach is necessary because each case discussion is brief, attributed to the high volume of cases that need to be reviewed. The results show that interviewees would welcome the possibility to use cBioPortal as a standalone solution, which is currently hampered by the need for parallel manual work. At the same time, the implementation of such an option would require a great deal of technical effort. The vision of PM4Onco is the deployment of MTB-cBioPortal as an exclusive tool for preparation and case discussion in MTBs, as a "one-stop shop”, so to speak.

Regarding visualization and more concrete layout options we will consider findings of Halfmann et al. who suggest a layout guided by (1) minimization of required actions and interfaces to improve the workflow, (2) support of information interpretation (e.g., through free arrangements and annotations, simultaneous visibility of all information, and the optimal use of space). In contrast to our approach of supporting both MTB preparation and presentation with a single system, they use two [50].

Schapranow et al. developed a software prototype to facilitate collaborative and virtual support for MTBs, focusing on enhancing the efficiency and effectiveness of case management [46]. During their study, existing clinical processes were analyzed, and users were surveyed to identify limitations and requirements, leading to the prototype’s evaluation with clinical experts. Their tool utilizes Kanban methodology for organizing patient cases, thereby positioning it primarily as a case management solution lacking a comprehensive integrated knowledge base. Many challenges identified in their work, such as time-consuming manual retrieval of information and the identification of similar patients, were also confirmed by our study. The functional and non-functional software requirements they derived can serve as a foundational framework for case-based MTB tool support. Our analysis further refines these requirements, particularly emphasizing the need for enhanced support through visualization techniques, thereby addressing the complex and dynamic nature of MTB case interpretation [46].

While tools like AMBAR [51], VMTB [52], and MTPpilot [47] focus on providing annotations from a variety of sources, offer complex genomics visualization, or in the case of MTB Assist [46] excel in supporting the MTB workflow, they provide no “one-stop shop”solution. In addition to limited use cases, applications focusing on supporting molecular tumor boards generally share a few common caveats. Data Integration proves to be especially challenging due to heterogeneous software infrastructures and a lack of standardized interfaces. While genomics data can generally be imported using established formats like VCF [53] or MAF [54], clinical data often must be entered through web interfaces, e.g., MTB-Report [55], or provided in a proprietary format. In cases like cBioPortal, the creation and validation process of these files may be supported through additional tools like the cbpManager [56]. Additionally, preferred annotation sources vary from clinician to clinician and require manual integration into existing tools—if possible, at all—as the database APIs are generally not standardized. While cBioPortal for example provides alteration annotations through OncoKB [57] and CIViC [58], clinicians still require time intensive manual literature search for the preparation of MTB cases to supplement the existing sources, which has often been communicated during the expert interviews. Overall, the current state of digital tools for molecular tumor boards especially underlines the need for automated integration of clinical data into MTB systems, extended documentation, and process support, solidifying the central findings of the requirements analyses. It also provides validity to the expansion of cBioPortal into a fully-fledged MTB software considering the absence of an existing holistic solution.

We give a brief outlook in which the preliminary work of the MII CDS set module "Molecular Genetic Findings Report" is taken up as a support for the FHIR resources implemented in cBioPortal, which are based on the CDS. The study aimed to establish a framework for integrating key data elements from German molecular genomics (MolGen) reports into electronic health records to enhance genomic and phenotype integration. Through collaboration within the German Medical Informatics Initiative, a core dataset of 76 data elements across six categories was developed, along with a FHIR specification comprising 16 profiles, including two additional profiles for FamilyMemberHistory and RiskAssessment resources. The mapping of MolGen report elements to ISO/TS 20428:2017 standard fields confirmed compliance, providing a template for standardizing genomic report data and facilitating its integration into electronic health records for clinical decision support [59].

We consider it useful and feasible to involve the interviewees in the process as future users. Despite their busy schedules, the participants were highly motivated to take part in the project. We believe that the prospect of a useful tool tailored specifically to their situation increased their willingness to participate. Many ideas were expressed about what interviewees would like to see in an adjusted version of cBioPortal. When asked what an "ideal system" would look like, they were quite aware of where the problems lie in the realizability of the requirements, namely technical feasibility and legal hurdles. We will therefore prioritize the user requirements according to their importance for general practitioners and technical feasibility. These prioritized user requirements will serve as the basis for the development of mockups and later the first prototype. In the next step, the user requirements are translated into system requirements and implemented technically. Mockups and prototypes are discussed and tested in several iterations with the participants.

## Conclusion

In our study, we observed a trend that with the implemented innovations the time required for the preparation of molecular tumor boards may be reduced, the quality of therapy suggestions may improve, and the developed solutions may provide better support than existing applications.

Through our comprehensive surveys and interviews, we identified and consolidated a set of critical requirements for the digital support of MTBs using cBioPortal. This collection highlights areas for improvement in data integration, visualization, and decision support not yet addressed. This work, grounded in clinical needs, aims to aid tool development to support MTB members in interpreting complex molecular data for personalized therapy recommendations. These findings provide a robust framework for ongoing development and extensions of MTB-cBioPortal. The focus is on creating solutions that are both technologically innovative as well as effective and user-friendly in their application to support and advance personalized medicine in oncology.

## Supplementary Information

Additional file 1. Questionnaire 1: MTB Preparation Team.

Additional file 2. Questionnaire 2: MTB Participants - variable team of specialists.

Additional file 3. Survey PM4Onco.

Additional file 4. Questionnaire: MTB participants – small separate survey. 

Additional file 5. Expert Interview guide.

Additional file 6. Summary results stage 1.

Additional file 7. Summary results stage 2.

Additional file 8. Code System.

Additional file 9. Summary results stage 3.

Additional file 10. Overview of cBioPortal.

## Data Availability

The data that support the findings of this study and the datasets generated and/or analyzed during the current study are not openly available due to reasons of sensitivity and the confidentiality promised to respondents at the time of consent and are available from the corresponding author upon reasonable request. Data are in controlled access data storage at Friedrich-Alexander-Universität Erlangen-Nürnberg, Department of Medical Informatics, Biometrics and Epidemiology, Medical Informatics, Erlangen, Germany.

## Abbreviations

API: Application Programming Interface
BAI: Binary Alignment Index
BAM: Binary Alignment Map
cBioPortal: CBio Cancer Genomics Portal
CDDS: Clinical Decision Support System
CDS: Core Dataset
CIViC: Clinical Interpretation of Variants in Cancer (knowledgebase)
CNA: Copy Number Alteration
CNV: Copy Number Variation
DNPM: German Network for Personalized Medicine
EHR: Electronic Health Record
FHIR: Fast Healthcare Interoperability Resources
HIS: Hospital Information Systems
MAF: Mutation Annotation Format
MII: Medical Informatics Initiative
MIRACUM: Medical Informatics in Research and Care in University Medicine
MSKCC: Memorial Sloan Kettering Cancer Center
MTB: Molecular Tumor Board
NGS: Next-Generation Sequencing
OncoKB: Oncology Knowledge Base
PM4Onco: Personalized Medicine for (4) Oncology
PROMs: Patient-Reported Outcome Measures
UC3: Use Case 3
UCD: User-Centered Design
VCF: Variant Call Format
VUS: Variants of Unknown Significance
ZPM: Centers for Personalized Medicine

## Footnotes

1 The German Network for Personalized Medicine (DNPM) is a cooperation of 26 university hospitals in Germany that have committed themselves to the mission of jointly improving the medical care and quality of life of people affected by advanced or rare cancers throughout Germany. To this end, Centers for Personalized Medicine (ZPM) will be established at all locations, which together with the existing ZPM in Freiburg, Heidelberg, Tübingen and Ulm will form the DNPM. The aim of the network is to provide patients with transparent and standardized access to personalized medicine. (https://dnpm.de/).

2 PM4Onco includes all four consortia of the MII, DIFUTURE (Data Integration for Future Medicine), HiGHmed, MIRACUM (Medical Informatics in Research and Care in University Medicine), and SMITH (Smart Medical Information Technology for Healthcare).

3 https://www.dekade-gegen-krebs.de/de/wir-ueber-uns/aktuelles-aus-der-dekade/_documents/PM4Onco.html

4 https://www.iso.org/standard/71952.html

5 IBM Corp. Released 2021. IBM SPSS Statistics for Windows, Version 28.0. Armonk, NY: IBM Corp.

6 https://github.com/nmalkin/plot-likert

7 MIRACUM Use Case 3 aims to streamline MTB case preparation, leveraging the existing MIRACUM-Pipe sequencing pipeline. This pipeline offers advanced variant annotation and facilitates the generation of interactive PDF reports and mutation data import into cBioPortal.(41).

8 https://www.clinicalgenome.org/data-sharing/clinvar/

9 KIS (Krankenhausinformationssystem) corresponds to HIS.

10 https://www.quickqueck.de/

11 https://bzkf.de/

12 Several biomarker subtypes have been identified according to their presumed applications. Understanding a single biomarker becomes much more complex when the interactions between several biomarkers are taken into account. For example: LDL cholesterol is related to the risk of cardiovascular disease and lower LDL cholesterol is better; higher systolic blood pressure is related to stroke and lower systolic blood pressure is better. (42).

13 https://www.uniklinika.de/die-deutschenuniversitaetsklinika/
