# Supplementary material for: Empowering personalized oncology: evolution of digital support and visualization tools for molecular tumor boards
Source: BMC Med Inform Decis Mak. 2025 Jan 16;25:29. doi: 10.1186/s12911-024-02821-8 (PMC11736948; doi:10.1186/s12911-024-02821-8)
Supplement: Supplementary file 2 — Additional file 2. Questionnaire 2: MTB Participants - variable team of specialists. [file 12911_2024_2821_MOESM2_ESM.docx]

Online-Survey

Evaluation of the molecular tumor board platform (MTB platform) developed as part of the MIRACUM medical informatics consortium

Dear survey participant,

We are delighted that you have decided to take part in today's survey on the topic "Evaluation of the Molecular Tumor Board Platform (MTB Platform) developed as part of the MIRACUM medical informatics consortium". The survey is being conducted by the coordinator of the MIRACUM consortium, the Chair of Medical Informatics at Friedrich-Alexander-Universität Erlangen-Nürnberg (FAU).

The survey will take about 15 minutes in total. You will be asked A. questions about the conventional way of working in the preparation of the molecular tumor board without the MTB platform, B. questions about the use of the MTB platform in the tumor board preparation and the acceptance of the platform and C. questions about yourself. The results of this survey provide us with important insights into the current state of development of the MTB platform and, if necessary, allow us to derive strategies and measures to improve the acceptance and use of the MTB platform in the future.

If you have any questions about the survey, please do not hesitate to contact Mr. Philipp Unberath, Tel.: +49 9131 85- 67788, E-Mail: philipp.unberath@fau.de.

You can view the detailed study information including the privacy policy here →Link Study information (see separate document). By answering this questionnaire, you consent to the anonymized storage and processing of the data in compliance with data protection regulations. The questionnaire will start as soon as you agree to participate in the survey.

Thank you very much for your time and support!

Your MIRACUM MTB team

I agree to the survey. →Link (continue to the next page)

I decline the survey. →Link (cancel the survey)

## Part A. Questions on the conventional / usual way of working without using the "MTB platform"

Please use the following questions to assess how you felt about **your previous /** usual way of working - i.e. preparing the Molecular Tumor Board (MTB) **without using the extended cBioPortal and without using the PDF report**:

| **Index** | **Working method before using the extended cBioPortal and the PDF report** | | | | | | |
| --- | --- | --- | --- | --- | --- | --- | --- |
|  |  | absolutely not satisfied | rather not satisfied | partial-part | rather satisfied | absolutely satisfied | not specified |
| A1 | Overall, how **satisfied**have you been **with your conventional practice** of preparing the MTB (searching for the required data, interpreting the data)? |  |  |  |  |  |  |
| A2 | Which **systems / applications / websites** have you used so far to **prepare for the MTB**(especially for searching for the relevant information)? | I didn't use any additional systems/applications/websites for the preparation of the MTB.   - So far, I have used the following systems / applications / websites for the preparation of the MTB: _________________________________ | | | | | |
|  |  | absolutely not supported | rather not supported | partial-part | rather supported | absolutely supported | not specified |
| A2.1 | **If you have used additional systems / applications / websites for MTB preparation:** **How well electronically supported** have you felt by these applications / databases / search functions **in the process of preparing the MTB** (searching for the required data, interpreting the data)? |  |  |  |  |  |  |
| A2.1.1 | **If you previously indicated "rather / absolutely not supported":** In your opinion, what were the **biggest obstacles and barriers** in preparing the MTB or using the systems / applications / websites? | Obstacles and barriers in the electronically assisted preparation of the MTB: ______________________________________ | | | | | |
|  |  | very low | rather low | medium | rather high | very high | not specified |
| A3 | In your opinion, how much **time has been spent so far** **searching for the data required** for interpretation? |  |  |  |  |  |  |
| A4 | How much **time** did it take you **on average to review a case** in preparation for the MTB? |  |  |  |  |  |  |
| A4.1 | - for **simple** cases: | estimated about ________________ minutes per case   - no information / don't know | | | | | |
| A4.2 | - for **moderately severe** cases: | estimated about ________________ minutes per case   - no information / don't know | | | | | |
| A4.3 | - for **complex** cases: | estimated about ________________ minutes per case   - no information / don't know | | | | | |
|  |  | very inaccurate / very incomplete | rather inaccurate / rather incomplete | partial-part | rather accurate / rather complete | very accurate / very complete | not specified |
| A5 | From **your point of view, how** accurate and complete **were the data needed to interpret a case** in preparation for the MTB? |  |  |  |  |  |  |
|  |  | very dissatisfied | rather dissatisfied | partial-part | rather satisfied | very satisfied | not specified |
| A6 | How **satisfied** have you been so far with **the clarity of the presentation of the data** for the case review in preparation for the MTB? |  |  |  |  |  |  |
|  |  | very insecure | rather insecure | partial-part | rather safe | very safe | not specified |
| A7 | How **sure have you been so** far that **you have made the most well-informed and, from your point of view, optimal interpretation for a case on the basis of the available data?** |  |  |  |  |  |  |

## Part B. Questions about the use of the MTB platform for case review and the acceptance of the MTB platform

### B1. Questions about using the MTB platform.

### For the following questions, please indicate **whether, how often and how extensively you have used the "extended cBioportal" and/or the "PDF report"** for the case review.

| **Index** | **Frequency and scope of use** | | | | | | |
| --- | --- | --- | --- | --- | --- | --- | --- |
| B1 | **How long** has the "extended cBioPortal" been available to you in your institution? | for ___________________ weeks   - no information / don't know | | | | | |
| B2 | **When** did you receive training **/ instruction** for the "extended cBioPortal"? | Not at all, I have not received any training or instruction on the "enhanced cBioPortal".  ________________________Wochen ago   - no information / don't know | | | | | |
|  |  | absolutely dissatisfied | rather dissatisfied | partial-part | rather satisfied | absolutely satisfied | not specified |
| B2.1 | **If you have received training/instruction: How satisfied** were you with the training/briefing? |  |  |  |  |  |  |
| B3 | **For how many cases** (estimated) **have** you **already used the "extended cBioPortal"**? | Absolutely not, I didn't use the extended cBioPortal.  Appreciated for _________________Fälle   - no information /don't know | | | | | |
| B3.1 | **If you previously indicated that you did not use the "enhanced cBioPortal",** what were the **reasons** for this? | Reasons for not using the "extended cBioPortal" (in bullet points):  __________________________________ | | | | | |
| B3.2 | **If you have previously indicated that you have used the "enhanced cBioPortal",** **which areas** of the system have you **used and how often**? | never | rare | occasionally | often | always | not specified |
| B3.2.1 | - Use of the extended **OnkoKB** |  |  |  |  |  |  |
| B3.2.2 | - Use of the **study search / Tab "ClicialTrialsGov"** |  |  |  |  |  |  |
| B3.2.3 | - **Documentation of the therapy recommendation** on the patient page / option "Therapy Recommendations" |  |  |  |  |  |  |
| B4 | **How long has** the **PDF report been** available **to you at your institution**? | for ___________________ weeks   - no information / don't know | | | | | |
| B5 | **For how many cases** (estimated) have you **already used the "PDF Report"?** | Not at all, I didn't use the PDF report.  Appreciated for _________________Fälle  no information /don't know. | | | | | |
| B5.1 | **If you have previously indicated that you do not want to use the "PDF Report"**: What were the **reasons** for this? | Reasons for not using the "PDF Report" (in bullet points):  __________________________________ | | | | | |
|  |  | never | Rare | occasionally | often | always | not specified |
| B5.2 | **If you previously indicated that you used the "PDF Report", how often** did you use the **hyperlinks** in the report? |  |  |  |  |  |  |

### B2. Questions about the current way of working with the MTB platform.

| **If you have NOT yet used the MTB platform, i.e. the "extended cBioPortal" and/or the "PDF report" to prepare for the MTB:** For all questions in this questionnaire block, please enter the answer option **"no answer"** and continue to the next questionnaire block. |
| --- |

Please use the following questions to assess how you **feel about** your current way of working **- i.e. the preparation of the Molecular Tumor Board (MTB) with the use of the extended cBioPortal and/or with the use of the PDF report:**

| **Index** | **Current working methods with "extended cBioPortal" and/or "PDF report"** | | | | | | |
| --- | --- | --- | --- | --- | --- | --- | --- |
|  |  | absolutely not satisfied | rather not satisfied | partial-part | rather satisfied | absolutely satisfied | not specified |
| B6 | How **satisfied** are you currently **with your practice** of preparing the MTB (searching for the required data, interpreting the data)? |  |  |  |  |  |  |
|  |  | absolutely not supported | rather not supported | partial-part | rather supported | absolutely Supported | not specified |
| B7 | **How well do** you currently feel supported by the "extended cBioPortal" and/or the "PDF report" **in the process of preparing the MTB** (search for the required data, interpretation of the data)? |  |  |  |  |  |  |
| B7.1 | **If you have previously indicated "absolutely not / rather not supported":** What do you think are the **biggest obstacles and barriers** when using the "extended cBioPortal" and/or "PDF reports"? | Obstacles and barriers when using the "extended cBio Portal" and/or "PDF reports": _______________________________________________ | | | | | |
|  |  | very low | rather low | medium | rather high | very high | keine Angabe |
| B8 | From your point of view, how much time is currently **required** to **search for the required data** for interpretation? |  |  |  |  |  |  |
| B9 | How much **time** do you currently need **on average to review a case** in preparation for the MTB? |  |  |  |  |  |  |
| B9.1 | - for **simple** cases: | estimated about ________________ minutes per case   - no information / don't know | | | | | |
| B9.2 | - for **moderately severe** cases: | estimated about ________________ minutes per case   - no information / don't know | | | | | |
| B9.3 | - for **complex** cases: | estimated about ________________ minutes per case   - no information / don't know | | | | | |
|  |  | very inaccurate / very incomplete | rather inaccurate / rather incomplete | partial-part | rather accurate / rather complete | very accurate / very complete | not specified |
| B10 | From **your point of view, how** accurate and complete is **the data currently required to interpret a case** in preparation for the MTB? |  |  |  |  |  |  |
|  |  | very dissatisfied | rather dissatisfied | partial-part | rather satisfied | very satisfied | not specified |
| B11 | How **satisfied** are you with the **clarity of the presentation of the data** for the case review in preparation for the MTB? |  |  |  |  |  |  |
|  |  | very insecure | rather insecure | partial-part | rather safe | very safe | not specified |
| B12 | How **sure** are you at the moment that you are making **the most well-informed and, from your point of view, optimal interpretation** for a case based on the available data? |  |  |  |  |  |  |

### B3. Questions about the acceptance of the MTB platform

| **If you use the MTB platform, i.e. you have NOT yet used the "extended cBioPortal" and/or the "PDF report" to prepare for the MTB: Please enter the answer option** "not specified" **for all questions in this questionnaire block** and proceed to the next questionnaire block. |
| --- |

Please use the following questions to assess how useful you find the **extended cBioPortal and the PDF report** for the preparation of the Molecular Tumor Board.

| **Index** | **Acceptance of the "extended cBioPortal" and the "PDF report"** | | | | | | |
| --- | --- | --- | --- | --- | --- | --- | --- |
|  |  | not useful at all | rather not useful | partial-part | rather useful | very useful | not specified |
| B13 | **Overall, how useful** do you think the **"enhanced cBioPortal"** is for your work? |  |  |  |  |  |  |
| B13.1 | **If you have previously indicated "rather / very useful": For which cases/situations,** do you think the **"extended cBioportal" offers the greatest added value**? | From my point of view, the "extended cBioPortal" would have the greatest added value especially for these cases / situations: ____________________________ (free text)   - no information / don't know | | | | | |
| B14 | How do you assess the **added value of the following functions of the "extended cBioPortal"** for your work? | absolutely no added value | rather no added value | partial-part | rather high added value | Absolute added value | not specified / not used |
| B14.1 | - Extended **OnkoKB** |  |  |  |  |  |  |
| B14.2 | - Direct **access to the study search** via the tab "ClicialTrialsGov" |  |  |  |  |  |  |
| B14.2 | - Possibility of **documenting the therapy proposal** |  |  |  |  |  |  |
|  |  | No, absolutely not. | No, not really. | I can't say. | Yes, probably. | Yes in any case. | not specified |
| B15 | Would you **prefer** to use the "enhanced CBioPortal" to your traditional way of working **without the "enhanced cBioPortal"**? |  |  |  |  |  |  |
| B15.1 | **If you answered "no" before,** what are the **reasons** for this? | Reasons for preferring the conventional way of working without an "extended cBioPortal" (in bullet points):  ______________________________________ | | | | | |
|  |  | not useful at all | rather not useful | partial-part | rather useful | very useful | not specified |
| B16 | **How useful** do you think the **"PDF Report"** is for your work overall? |  |  |  |  |  |  |
| B16.1 | **If you have previously indicated "rather / very useful": For which cases/situations,** do you think the **"PDF report" offers the most added value**? | - From my point of view, the "PDF report" would have the greatest added value especially for these cases / in these situations: _______________________________________________ - no information / don't know | | | | | |
| B17 | How do you assess the **added value of the following functions / representations of the "PDF Report"** for your work? | absolutely no added value | rather no added value | partial-part | rather high added value | absolute added value | not specified / not used |
| B17.1 | - **Hyperlinks** |  |  |  |  |  |  |
| B17.2 | - Presentation of the **ACMG classification and sorting of the top mutations** |  |  |  |  |  |  |
| B17.3 | - Summary of **key findings on the first page** of the report |  |  |  |  |  |  |
| B17.4 | - Name of **cancer genes** (oncogenes and tumor suppressor genes) |  |  |  |  |  |  |
| B17.5 | - **Mutation Signatures** |  |  |  |  |  |  |
|  | - Data of **TMB, MSS, HRD, tumor cell content (bioinformational), diploids** |  |  |  |  |  |  |
|  |  | No, absolutely not. | No, not really. | I can't say. | Yes, probably. | Yes in any case. | Not specified |
| B18 | Would you prefer to **use the "PDF Report" to your conventional way of working** without the "PDF Report"? |  |  |  |  |  |  |
| B18.1 | **If you answered "no" before**, what are the **reasons** for this? | Reasons for preferring the conventional way of working without a "PDF report" (in bullet points):  ______________________________________ | | | | | |

## Part C. Personal Questions

Finally, we would like to ask you to answer a few **questions about yourself**. They are very supportive in the evaluation of the survey.

| **Index** | **Personal details** | |
| --- | --- | --- |
| C1 | Your **gender**? | - male - female - divers - not specified |
| C2 | Your **age**? | Age in years: ______________ |
| C3 | What **specialization** do you belong to? | - Pathology - Haematology / Onkology - Systemmedizin - Bioinformatik - Medizininformatik - Other: ____________________ (Free text) - not specified |
| C4 | **How often** does the **MTB take place in your institution**? | - ______________ times a month - not specified |
| C5 | **How often do you take part in the MTB?** | - rare - occasionally - often - always - not specified |
| C6 | **How many cases do you handle on average** in preparation for the MTB? | - estimated slightly ____________________ cases per MTB - no information / don't know |
| C7 | **What percentage of the cases** you prepare for the MTB are...: |  |
| C7.1 | - ...**simple**? | - estimated about _____________ percent of cases - no information / don't know |
| C7.2 | - ...**moderately difficult**? | - estimated about _____________ percent of cases - no information / don't know |
| C7.3 | - …**complex**? | - estimated about _____________ percent of cases - no information / don't know |

A questionnaire cannot always address all aspects. There is room for your individual comments here. Is there anything else you would like to draw our attention to?

|  |
| --- |

**We would like to thank you very much for your valuable time and support of our work!**
