# Supplementary material for: Empowering personalized oncology: evolution of digital support and visualization tools for molecular tumor boards
Source: BMC Med Inform Decis Mak. 2025 Jan 16;25:29. doi: 10.1186/s12911-024-02821-8 (PMC11736948; doi:10.1186/s12911-024-02821-8)
Supplement: Supplementary file 3 — Additional file 3. Survey PM4Onco. [file 12911_2024_2821_MOESM3_ESM.docx]

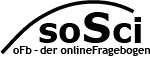
 Survey PM4Onco

PM4ONCO-AP5 → Base

17.01.2024, 14:13

# Page 01

Dear survey participant,

We are very pleased and thank you very much for deciding to participate in today's survey on the topic of "*Requirements Assessment for the Further Development of the Molecular Tumor Board Platform (MTB Platform)"* as part of the MII project
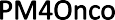
 . The survey will be conducted by the coordinator of the

PM4Onco Work Package 5: Visual Analytics, the Chair of Medical Informatics at Friedrich-Alexander-Universität Erlangen-Nürnberg (FAU).

Procedure & Duration

The interview takes about 15 minutes in total. You will be given:

A. Questions about the conventional way of working in the preparation of the Molecular Tumor Board,

B. Questions on requirements for visualization of the data in the MTB platform and

C. Questions about yourself.

The results of this survey provide us with important insights into your needs and requirements regarding the further development of the MTB platform. They also allow us to derive requirements for the development of enhancements for improved visualization of MTB case data to support case interpretation and thus improve the acceptance and use of the MTB platform in the future.

Further information

If you have any questions about the survey, please do not hesitate to contact Dr. Philipp Unberath, phone: +49 173 3735424, e-mail: philipp.unberath@fau.de.

You can view the detailed study information including the privacy policy here. By answering this questionnaire, you consent to the anonymized storage and processing of the data in compliance with data protection regulations. The questionnaire will start as soon as you agree to participate in the survey.

Thank you very much for your time and support!

Your PM4Onco AP5 Team

# Page 02

**A**

**A001**


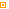


## Overall, how satisfied have you been with your traditional practice of preparing the MTB (search

**of the data needed for interpretation of the data)?**


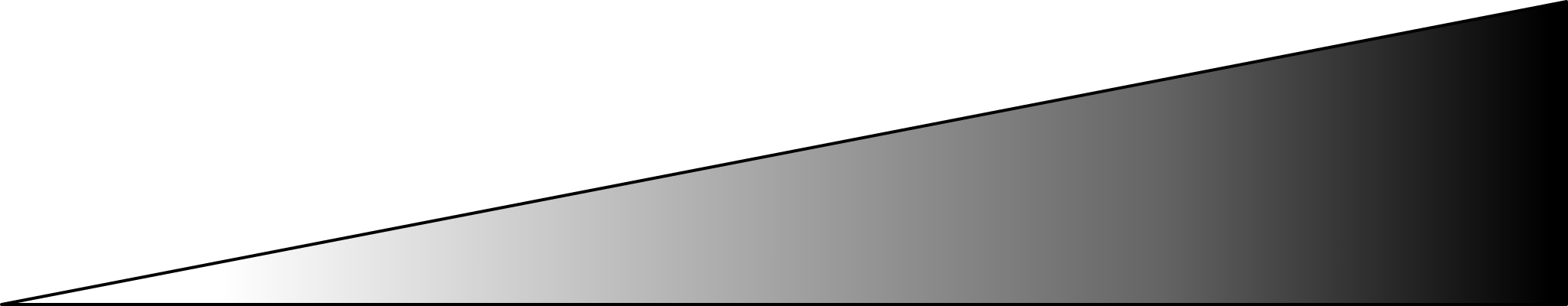


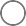


not specified


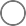

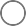

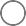

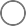

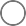


absolutely not satisfied

rather not satisfied

partial-part rather satisfied absolutely satisfied

## Which systems/applications/websites have you used so far for the preparation of the MTB (especially for searching for the relevant information)?


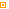


**A002**


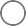
 I do not use any systems / applications / websites for the preparation of the MTB.


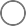
So far, I have used the following systems / applications / websites for the preparation of the MTB:

**A003**

## If you have used additional systems/applications/web pages for MTB preparation: How well have you felt electronically supported by these applications / databases / search functions in the process of preparing the MTB (search for the required data, interpretation of the data)?


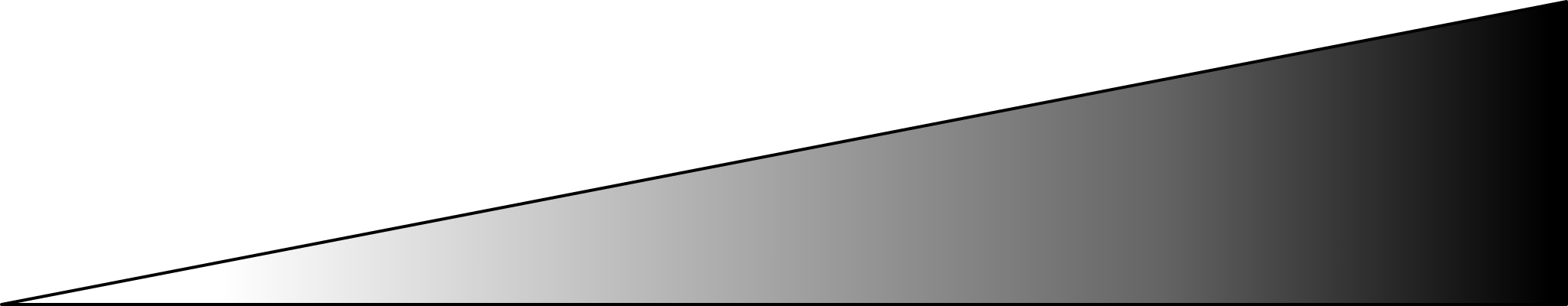


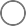


not specified


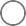

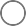

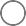

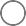

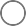


absolutely not supported

rather not supported

partial-part rather supported absolutely Supported

**A004**

## If you previously stated "rather/absolutely not supported": What do you think were the biggest

**obstacles and barriers in the preparation of the MTB or the use of the systems / applications / websites?**

Obstacles and barriers in the electronically supported preparation of the MTB:

## Have you worked with the cBioPortal platform before?

**A005**

(Multiple selections possible)


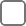
 No

Yes, with the public version in research context


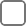


- Yes, with the public version to support data interpretation in the MTB


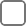
 Yes, with its own local version in a research context


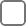

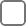
 Yes, with its own local version for the MTB

Yes, in the following context:

## From your point of view, how much time has been spent so far in finding the data needed for interpretation?


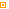


**A006**


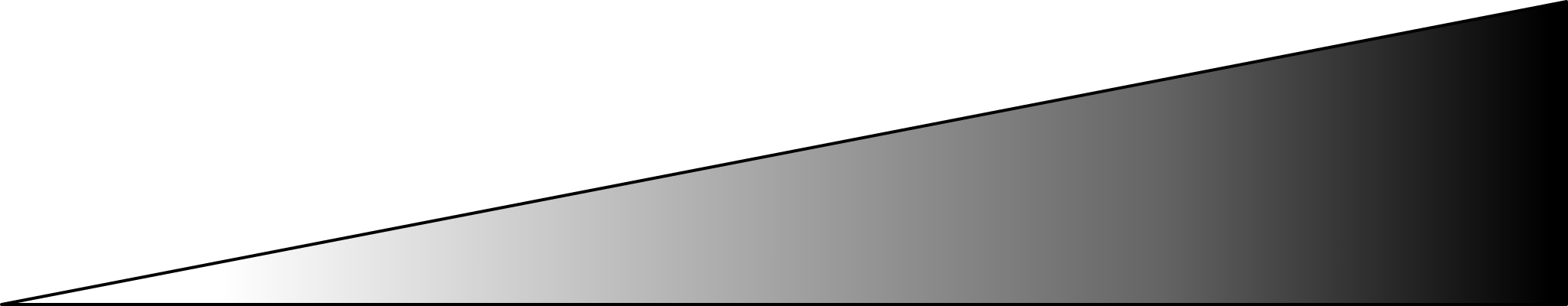


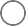


not specified


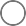

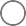

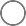

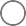

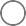


very low rather low medium rather high very high

**A007**


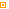


## How much time did it take you on average to review a case in preparation for the MTB?

For simple cases, estimated about minutes
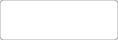
 per case

For moderate cases, estimated about
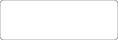
 minutes per case

For complex cases, estimated about minutes
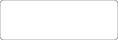
 per case


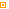


**A008**

## In your opinion, how accurate and complete was the data required to interpret a case in preparation for the MTB?


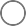

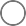

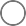

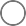

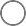

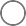


very inaccurate / very incomplete

rather inaccurate / rather incomplete

partial-part rather accurate / rather complete

very accurate / very complete

not specified

**A009**


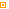


## Do you use or take into account Patient Reported Outcomes (PROMs) for the interpretation of a case?


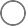
 no, not in any case

- partly – depending on the case


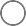
 yes, in each case


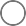
no information / don't know

**A010**


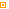


## How satisfied have you been so far with the clarity of the presentation of the data for the case review for the


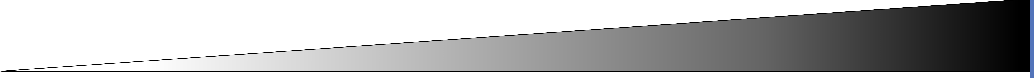
**preparing for the MTB?**


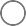


not specified


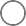

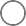

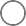

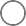

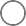


very dissatisfied

rather dissatisfied

partial-part rather satisfied very satisfied

##
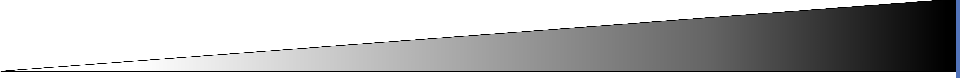
How sure have you been so far that you have made the most well-informed and, from your point of view, optimal interpretation for a case on the basis of the available data?


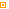


**A011**


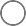


not specified


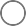

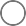

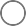

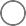

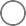


very insecure rather insecure partial-part rather safe very safe

##
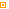
What systems/methods have you used so far in MTB to visualize data on MTB cases?

**So far, I have used the following systems/methods for visualizing this data from MTB cases:**


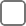

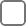

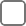

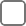

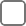

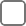

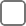

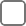

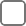
Clinical data:

Genomic variants:

CNA/CNV:

Gene expression:

(Novel) Complex biomarkers:

History:

Patient Reported Outcome Measures (PROMs):

Other:

I don't use any systems/methods for visualizing the data of MTB cases.

# Page 03

**B**

##
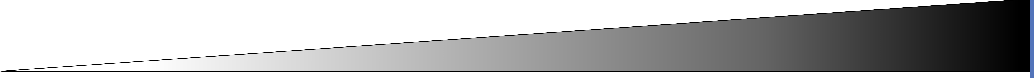
In general, how do you estimate your need for additional data visualization methods in MTB?


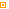


**one?**

**B001**


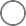


not specified


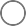

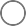

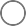

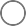

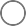


very little need

little need partial-part hjigh demand very high

Need

**B002**

1. **If you previously specified "high/very high demand": For which cases/situations, do you see**

**Is there a specific need for additional visualization methods?**

**From my point of view, the greatest need for visualization methods is mainly for these cases / situations:**

**B003**

## In the PM4Onco project, we plan to display collected Patient Reported Outcomes (PROMs) in the MTB or to

**to visualize. What added value would such an ad have for you?**

No added value: From my point of view, I don't really need that in MTB for a case discussion.


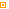


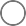
 Early detection of changes during a patient


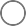

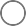
 Making a more targeted and better therapy decision Other added value:


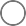
not specified


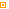


**B004**

## How do you assess the need for new visualization methods for the following data elements?

Clinical Data

very little need

little need

partial-part

High demand

Very high demand

*not specified*

genomic variants

very little need

little need

partial-part

High demand

Very high demand

*not specified*

CNA/CNV

very little need

little need

partial-part

High demand

Very high demand

*not specified*

Gene expression

very little need

little need

partial-part

High demand

Very high demand

*not specified*

(novel) complex biomarkers

very little need

little need

partial-part

High demand

Very high demand

*not specified*

History

very little need

little need

partial-part

High demand

Very high demand

*not specified*

Patient Reported Outcome Measures (PROMs) for a single patient

very little need

little need

partial-part

High demand

Very high demand

*not specified*

Patient Reported Outcome Measures (PROMs) of a Norm/Reference Population

very little need

little need

partial-part

High demand

Very high demand

*not specified*

## For which additional data elements do you see a need for new visualization methods?


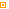


**B005**


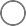
In my view, there is a need for new visualization methods for the following data elements:


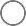
no information / don't know

## What specific visualization methods are you aware of that support (or could support) the processes of the MTB (e.g. data interpretation)?


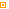


**B006**


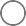
These specific visualization methods can support the processes of the MTB:


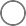
no information / don't know

## Which data elements or visualization methods do you use in particular when preparing / creating therapy recommendations?


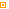


**B007**

- I use these data elements or visualization methods when preparing / creating therapy recommendations:


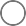
no information / don't know

## If you previously specified "high/very high need": For what cases/situations do you see specific need for methods to assess patient similarity and compare similar patients?


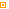


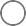


In my opinion, the greatest need for methods for assessing patient similarity is primarily for these cases / situations:


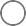
no information / don't know

**B011**


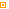


## What specific methods for assessing patient similarity or comparing similar patients who support (or could support) the processes of the MTB (e.g. data interpretation)?

These concrete methods for assessing patient similarity and comparison of similar patients could support the processes of the MTB:

no information / don't know

## Which projects / plans are you aware of at your own location and across locations that are currently developing visualization methods for the MTB?

**To**

**B012**

I am familiar with these projects that develop visualization methods for MTB:

no information / don't know

**B008**

## In your opinion, how important is it to customize the user interface and visualizations according to your own

**needs?**

not specified

absolutely not important

not important partial-part important absolutely important

**B009**

## How do you estimate your need for methods to assess patient similarity and comparison similar patients in the MTB?

not specified

very little need

little need partial-part high demand very high

need

# Page 04

**C**

## Your gender?

**C001**

- Male

Female Diverse

not specified

## Your age?

**C002**

Age in years:

## Your position?

**C003**

- Student in Practical Year (PJ)
- Assistant Physician
- Specialist Senior
- Physician
- Senior Physician
- Medical Director

Other:

not specified

## Which field of study is yours?

**C004**

(Molecular/Clinical) Pathology

Hematology / Oncology

Systems Medicine Systems Biology Molecular Biology Human Genetics

Bioinformatics

Medical Informatics Other:

not specified

**C005**

## How often does the MTB take place in your institution?

time in a month

## How often do you take part in the MTB?

**C006**

rare

occasionally often

always

not specified

## How many cases do you handle on average in preparation for the MTB?

**C007**

estimated about cases per MTB

## What percentage of the cases you prepare for the MTB are

**C009**

simple?

Moderate?

complex?

1. **A questionnaire cannot always address all aspects. There is room for your individual comments here. Is there anything else you would like to draw our attention to?**

**C010**

# Last Page

**Thank you for participating!**

We would like to thank you very much for your valuable time and support of our work!

Since this questionnaire cannot cover all relevant aspects, we would like to invite you to participate in a focus group interview. The aim of this additional survey is to gather a broad expertise in the field of MTB at your location to jointly identify and discuss additional requirements for visualizations for MTB. The link to the appointment can be found in the same e-mail in which the link to this questionnaire was contained.

Your answers have been saved; you can now close the browser window.

[Dr. Philipp Unberath](mailto:philipp.unberath@fau.de)  – 2023
