# Supplementary material for: Empowering personalized oncology: evolution of digital support and visualization tools for molecular tumor boards
Source: BMC Med Inform Decis Mak. 2025 Jan 16;25:29. doi: 10.1186/s12911-024-02821-8 (PMC11736948; doi:10.1186/s12911-024-02821-8)
Supplement: Supplementary file 5 — Additional file 5. Expert Interview guide. [file 12911_2024_2821_MOESM5_ESM.docx]

**Personalized Medicine for Oncology (PM^4^ Onco) Requirements for Visualization Solutions for MTB**

**Expert Interview Guide**

**1. General information**

Participating:

Implementation team: Philipp Unberath, Dominik Böhm, Cosima Strantz

| Expert | Location | Role in MTB |
| --- | --- | --- |
|  |  |  |
|  |  |  |
|  |  |  |
|  |  |  |

Duration:

The duration of the interview is **approx. 60 minutes** (extension of 30-60 minutes possible).

Organizational:

The interviews will be **recorded**. This is **exclusively for the subsequent transcription** and summary of the results of the interview. The **results of the interviews are only summarized and published**, so that **no conclusions can be drawn about individual persons**. Records and logs are **destroyed after evaluation**.

☐ All participants have **signed the** privacy policy

What it's about:

The PM4Onco project aims to **strengthen personalized medicine in oncology**.

In the specific context of work package 5, the focus is on the **development of innovative visualization solutions** for the molecular tumor board. These solutions are expected to play a **central role in decision-making** and strategy development for individualized treatment plans.

The **requirements assessment forms a crucial basis** for the development of visualization solutions that are specifically tailored to the **needs of the Molecular Tumor Board**. It is divided into two parts:

1. (Quantitative) Anonymized online survey by means of questionnaires (completed)
2. (Qualitative) Survey by means of expert interviews (now)

**2. Getting Started**

- Short introduction and **description of the MTB wheel**
- Who answered the **questionnaire**?

A brief overview of the **current workflow** and the tools used:

- What specific tools are currently being used and why?
  - Data analysis
  - Data interpretation (diagnostics / therapy recommendation)
  - Follow-up / Documentation MTB
  - Are there any aspects or features of these **tools** that you feel are **exemplary** or **could be improved**?
- What are the **special features** of the current **process**?
  - What works particularly well / badly?
  - Are there missing functionalities in the tools used?
  - What could be made simpler/more efficient?

**3. Challenges and need for support**

- What **challenges** do you face when preparing and executing MTBs and how **do they influence your work**?
- What kind of **technical or procedural support** would help you overcome these challenges?
- Can you give **examples** where such support would be particularly helpful?
- How do you think the **time spent** preparing and executing MTBs could be **reduced**?
- Could steps  **be automated?**

**4. Requirements for Visualization Methods**

- What **types of data** (e.g., genomic data, clinical data, patient-reported outcomes) do you think need **improved visualization methods**?
  - Genomic data
  - CNV/CNA
  - Gene expression
  - Clinical Data
  - PROMs
  - History
- Are there specific data types or information that are **not sufficiently visualized** in current systems?
- What challenges do you face when **visualizing complex or large data** (complex biomarkers)?
- What **features** could be helpful in visualizing this data (e.g., interactive elements, filter options, customization options)?
- To what extent should visualization tools be **interactive** to support your work process?
- What **customization options** (e.g. selection of data points, adjustment of axes) would be beneficial for you?
- How is the **search for similar patients** currently mapped and how could this be improved?

**5. Integration**

- How would you ideally integrate visualization tools **into your daily workflow**?
- Are there **specific situations or scenarios** where improved visualization methods would be particularly useful?

**6. Outlook**

- **Make a wish**: Do you have any ideas or wishes for future-oriented visualization methods?
- Are there any other comments or ideas you'd like to share?
- Do you still want to be contacted?
  - (Intermediate) Rounds of Requirements Gathering
  - Evaluations: formative / summative

**7. Acknowledgements**

We would like to thank you very much for the time invested and the support of the project!

If you have any questions or other concerns, please feel free to contact us by e-mail at any time.
