# Supplementary material for: Empowering personalized oncology: evolution of digital support and visualization tools for molecular tumor boards
Source: BMC Med Inform Decis Mak. 2025 Jan 16;25:29. doi: 10.1186/s12911-024-02821-8 (PMC11736948; doi:10.1186/s12911-024-02821-8)
Supplement: Supplementary file 6 — Additional file 6. Summary results stage 1. [file 12911_2024_2821_MOESM6_ESM.docx]

### Summary results stage 1

The main challenges encountered in electronically supported preparation for the MTB without using the MTB platform include:

- Lack of user interfaces, necessitating manual input of patient data into cBioPortal, which is very time consuming.
- Databases not tailored to the needs of the MTB (providing therapy decision support, (pre)clinical data, drug labeling, and ongoing clinical trials). OncoKB still offers the best solution.
- Lack of options for collaboration, which complicates the search for specific mutations due to ambiguous nomenclature.
- Lack of automatic linking of variants to a suitable platform for evaluation and further analysis.
- Evaluation of the detected variants with those observed in the cited therapy stratification studies, which requires manual integration of information from different websites, resulting in a significant time investment.
- Time-consuming and confusing processes, as information is only partially validated or incomplete.
- Searching across multiple platforms or through repeated entering the system, and navigating multiple submenus take a lot of time.

Regarding the extended cBioPortal, key reasons for its underutilization include:

- The lack of automatic data transfer for mutations and clinical data, resulting in extra work during MTB preparation.
- The absence of external or mobile access.

Integrating the extended cBioPortal into existing systems could enhance its usefulness, providing added value through its functionalities compared to conventional methods:

- The integration of the extended cBioPortal into existing systems is rated as ‘rather good’, on 5-point Likert scales, ranging from ‘very poor’: "I can only access the "extended cBioPortal" with great difficulty and in several steps." to ‘very good’: "The extended cBioPortal has been integrated very well and is easy and quick to access."
- The "extended" cBioPortal is rated as ‘rather useful’ to ‘very useful’ on 5-point Likert scales, ranging from ‘not at all useful’ to ‘very useful’ and the added value of the individual functions/areas is given as ‘rather high added value’ to ‘absolute added value’, on a scale from ‘absolutely no added value’ to ‘ab-solute added value’. The "extended cBioPortal" would be preferred to the conventional way of working (cBioPortal without MIRACUM-specific extensions)

Reasons for not using the PDF report:

- MTB documentation takes place in electronic patient file without PDF findings;
- Information is already available online; PDF reports from pathology and bioinformatics are already available.

Greatest added value of the PDF report:

- Overall, the PDF report is rated as ‘rather useful’ to ‘very useful’ (on 5-point Likert scales from ‘not at all useful’ to ‘very useful’); the added value of the individual functions/area is given as ‘rather high added value’ to ‘absolute added value’ (from ‘absolutely no added value’ to ‘absolute added value’);
- Free text responses were the following: annotations; better interface communication; possibility of integration in MTB for joint case discussion; access from external / mobile, outside the hospital network; very good overview of molecular analyses and basic data; hyperlinks are very good.

Summarized answers to the research question of the study:

Q1: variable: degree of process improvement; tends to: yes:

- Reduction of (perceived) time spent with MTB platform; tendency of time spent from ‘rather high’ to ‘medium’ (range: ‘very low’ to ‘very high’;
- Tendency of reduction of perceived time effort for all kind of cases (simple, medium, complex).

Q2: variable: degree of result improvement; tends to: yes:

- Preparation team: no change; respondents were already "somewhat satisfied" previously;
- Specialist team: minimal improvement; tending from ‘partly-somewhat’ to ‘very accurate / completely; very satisfied / very confident’. range

Q3: variable: degree of support for one's own work; tends to yes:

- - Respondents tend to feel better supported with "extended" cBioPortal and PDF report;
  - The enhanced cBioPortal and PDF report would be preferred over the traditional way of working (cBioPortal without MIRACUM-specific enhancements / no PDF report);

Q4: Variable: perceived added value of the MTB platform; tends to yes:

- - The "extended" cBioportal is rated as ‘rather useful’ to ‘very useful’; the added value of the individual functions / area is stated as ‘rather high added value ‘absolute added value’; (ranges see above);
  - The PDF report is rated as ‘rather useful’ to ‘very useful’; the added value of the individual functions / area is given as ‘rather high added value’ to ‘absolute add-ed value’; (ranges see above);
  - Free-text response covered the topics: need for training, need for integration with existing systems (due to lack of interfaces and the necessity of redundant documentation), extension of functionality: links to other important databases, interpretation aid for fusions, result presentation of fusions and molecular pathology results.
