# Supplementary material for: Empowering personalized oncology: evolution of digital support and visualization tools for molecular tumor boards
Source: BMC Med Inform Decis Mak. 2025 Jan 16;25:29. doi: 10.1186/s12911-024-02821-8 (PMC11736948; doi:10.1186/s12911-024-02821-8)
Supplement: Supplementary file 7 — Additional file 7. Summary results stage 2. [file 12911_2024_2821_MOESM7_ESM.docx]

### Summary results stage 2

Below, we report the results obtained from the web-based surveys and interviews. We conducted the first round of surveys for the preparation teams with a total of 25 participants (11 female, 7 male, 7 not specified). Representatives of the following disciplines were involved: Hematology/Oncology (9+2), Molecular biology (4), Human genetics (2), Gynecology (2), Systems medicine and Bioinformatics (2), Neurosurgery (1), and not specified (7). Respondents were on average 37 years old (range 25 to 50 years). They had been members of the MTB for a mean time of three years. The MTB took place on average twelve times per month with an average of nine cases per session. We conducted the interviews with a total of 9 participants (7 female, 2 male) with positions in: Hematology/Oncology (5), Coordinator (2), Resident (1), Neuroscientist (1).

Requirements from Quantitative Analysis

The evaluation of the survey data identified the following results relevant to our research question:

- Variety of tools used: The survey revealed that a variety of sources such as Pub-Med (14/25), cBioPortal (9/25), Google Search or Google Scholar (9/25) and ClinVar (7/25), (not summative) were used to prepare the MTB. This reflects the interdisciplinary and information-intensive nature of the preparation process.
- cBioPortal: Almost all users have already gained experience with the use of cBioPortal (22/25), primarily as a local application to support the preparation of the MTB (10/25) and for use in research (7/25, (not summative);
- Satisfaction versus support: Although users are generally satisfied with the conventional practice of preparing the MTB, they tend to feel insufficiently supported by the current systems in terms of electronic support and the efficiency of the search process;
- Time spent on interpretation: consistent feedback was that the time spent on interpreting data is perceived as rather high or very high, which underlines the need for more efficient analysis tools. Simple cases took an average time spent of 18 minutes, moderate cases of 36 minutes, and severe cases about 77 minutes. Rating scale;
- Accuracy and completeness of data: Users predominantly rated the available data to them as accurate and complete and can generally find an optimal interpretation for themselves based on this. This shows the high level of trust in the data quality, but also the need for supporting tools for data analysis;
- Visualization formats used: PowerPoint slides/PDF and downstream cBioPortal dominate the methods currently used to visualize data. Zahlen PowerPoint/PDF presentations are seen as a simple way of manually combining and presenting a variety of different data;
- Patient-Reported Outcome Measures (PROMs): Almost no users (3/25) stated that they currently already use a system that visualizes PROMs for use in the context of the MTB. This underlines the remaining need for development, especially in contrast to the existing tools for other visualization tasks. At the same time, how-ever, only a very small proportion (3/25) of users’ report having used PROMs for the interpretation of cases at all;
- Need for additional visualization methods: There is a clear need (high to very high need: 10, partial need: 9) for additional visualization methods, especially for molecular biology data. This underlines the need to develop specialized and advanced visualization options. In addition, need for additional data visualization methods in MTB was assessed as high or very high by 10 of 20 participants, 9 appraised a party need, only 1 indicated low need, 5 were not specified;
- Customizability of the user interface: The customizability of the user interface is considered (absolutely) important by 15 out of 25 respondents. A flexible and user-friendly interface is crucial for the efficiency and effectiveness of the software.
