# Supplementary material for: Empowering personalized oncology: evolution of digital support and visualization tools for molecular tumor boards
Source: BMC Med Inform Decis Mak. 2025 Jan 16;25:29. doi: 10.1186/s12911-024-02821-8 (PMC11736948; doi:10.1186/s12911-024-02821-8)
Supplement: Supplementary file 8 — Additional file 8. Code System. [file 12911_2024_2821_MOESM8_ESM.pdf]

Code System (stage 3)

Code System	Frequency
Code System	
Solutions, visions	18
Current workflow	49
Databases	6
Pathological findings	8
Tools	10
Time required	25
Visualization	20
History data	11
PROMs	8
Patient like me	8
complex biomarkers	7
cBioPortal	72
View	5
Annotation sources	8
Standard or customized solution	7
Evidence	6
visionary features	7
Interfaces	9

Missing data types	12
Missing features	18
Media breaks	10
Study search	32
Congress paper	2
Literature databases	18
PubMed	12
MTB process	48
Tumor documentation	8
Role in MTB	7
MTB presentation	16
Case presentation	16
MTB platform	17
PowerPoint	5
Need for support	7
Automation	5
Data types	18
Support	35
Integration	22
cBioPortal	10
Study search	12
Documents	31

Paper form	7
own representation	8
own lists	7
PDF	9
