# Supplementary material for: Empowering personalized oncology: evolution of digital support and visualization tools for molecular tumor boards
Source: BMC Med Inform Decis Mak. 2025 Jan 16;25:29. doi: 10.1186/s12911-024-02821-8 (PMC11736948; doi:10.1186/s12911-024-02821-8)
Supplement: Supplementary file 9 — Additional file 9. Summary results stage 3. [file 12911_2024_2821_MOESM9_ESM.pdf]

Summary results stage 3 (requirements list)

Data Integration and Management:

- Ability to integrate diverse data sources, including electronic health records (EHRs), molecular test results, imaging data, and clinical notes.
- Secure storage and caring of sensitive patient data, ensuring compliance with privacy regulations such as Federal Data Protection Act (Bundesdatenschutzgesetz, BDSG), Medical Devices Act (Medizinproduktegesetz, MPG)

Molecular Data Analysis:

- Processing molecular data, including genomic sequencing results, tumor mutational profiles, and biomarker assessments electronically from pathology.
- Visualization tools to interpret molecular data effectively, such as interactive genomic maps and mutation frequency plots.

Clinical Decision Support:

- Algorithms and decision support systems to interpret molecular findings and recommend personalized treatment options based on evidence-based guidelines and clinical trials data. (This indeed was not mentioned by the interviewees but completes this requirement.)
- Integration with knowledge bases and databases of cancer mutations, drug interactions, and therapeutic targets, clinical trials.

Collaborative Tools:

- Real-time communication and collaboration feature to facilitate discussions among multidisciplinary team members, including oncologists, pathologists, genetic counselors, and researchers regarding case-information, shareable across sites.
- Support for virtual meetings, video conferencing, and asynchronous communication to accommodate remote participants and busy schedules. (This indeed was not mentioned by the interviewees but completes this requirement.)

Security and Compliance:

- Robust authentication and authorization mechanisms to control access to sensitive patient data and ensure data privacy and security.
- Compliance with relevant healthcare regulations (e.g., …), and regular security audits and testing.

Usability and User Experience:

- Intuitive user interface design optimized for healthcare professionals with di-verse backgrounds and levels of technical expertise.
- Support for customizable workflows, role-based access controls, and personalized user preferences to streamline the MTB process.

Scalability and Performance:

- Scalable architecture to support growing volumes of molecular data and in-creasing numbers of MTB participants and cases.
- High availability, fault tolerance, and performance optimization techniques to ensure responsiveness and reliability under heavy usage.

Interactive Data Visualization:

- Support for interactive charts, graphs, and plots to visualize molecular data.
- Ability to zoom, pan, and filter data.

Temporal Visualization:

- Visualization of longitudinal molecular data to track changes in tumor evolution, treatment response, and disease progression over time.
- Time-series plots, heatmaps, or animated visualizations to illustrate temporal dynamics of molecular alterations and treatment effects.

Training and Support:

- Comprehensive user training materials, tutorials, and documentation to onboard new users and maximize utilization of the platform.
- Responsive customer support and helpdesk services to address technical is-sues, answer user queries, and provide ongoing assistance.
