# Supplementary material for: Empowering personalized oncology: evolution of digital support and visualization tools for molecular tumor boards
Source: BMC Med Inform Decis Mak. 2025 Jan 16;25:29. doi: 10.1186/s12911-024-02821-8 (PMC11736948; doi:10.1186/s12911-024-02821-8)
Supplement: Supplementary file 10 — Additional file 10. Overview of cBioPortal. [file 12911_2024_2821_MOESM10_ESM.docx]

## Overview of cBioPortal

### cBioPortal – an Open-Source Platform

The open-source tool cBioPortal is a web-based platform for exploring and visualizing multidimensional cancer genomics data, hosted by the Memorial Sloan Kettering Cancer Center (MSKCC). This public instance enables researchers access to a collection of manually curated cancer studies and detailed analyses. Furthermore, cBioPortal offers the option of setting up local instances and integrating them into an inhouse IT infrastructure. This allows users to load their datasets into the local platform and to analyze potentially sensitive data in compliance with data protection regulations (1,2).

The most important enhancements since stage 1 were two new tabs in the patient view in cBioPortal (1) the automatic search for clinical trials based on genomic and clinical patient data by integrating ClincalTrials.gov, since cBioPortal does not natively provide automatic matching of a patient's genomic profile with relevant clinical trials. Studies suggested in the tab can be adopted, but require further manual research (3). And a (2) tab is the structured and standardized documentation of treatment recommendations (4). Minor enhancements and customizations relate to the approval status of drugs by the European Medicines Agency (EMA) directly into the available Oncology Knowledge Base (OncoKB) (3) annotation, import and display of LoH mutations and the handling of internal PDF documents (4). Within PM4Onco, the goal of defining a harmonized application and extension for Personalized Oncology (PersOnco) in the national core dataset (CDS)^[[1]](#footnote-1)^ of the MII, based on the DNPM, the MII CDS and the nNGM^[[2]](#footnote-2)^ dataset especially for molecular pathology based on the published FHIR profiles.^[[3]](#footnote-3)^

### MTB-cBioPortal

The recent release of MTB-cBioPortal (2024q1) is based on cBioPortal v6.0.4. Beside the new features added by the upstream cBioPortal project, this release is focused on the deployment process and the compatibility with HIS. One step to simplify the setup is the migration of the user authentication using Keycloak from SAMLv2 to Open ID Connect, as productive setups also include HTTPS transport encryption (5). To ease the integration with HIS, the therapy recommendations are now linked to an order id, so they can be linked correctly in the electronic health record. Another necessary change was the extension of the evidence level documentation, to achieve conformance with the guidelines of the German Centers for Personalized Medicine.^[[4]](#footnote-4)^

### System Specifications

CBioPortal conduces to two primaries fields of application: translational research and MTBs. Originally developed as a research tool, cBioPortal organizes data into studies, making it accessible for researchers to analyze published studies and explore data relationships. It has gained popularity for its ability to streamline complex raw data analysis through built-in evaluations (6,7). In the MTB context, clinics leverage cBioPortal to diagnose and treat patients based on molecular test results. While developers from MSKCC have made custom adaptations, such as linking mutations to drug information via OncoKB, further local adaptations are needed. ^[[5]](#footnote-5)^ The public version lacks standardized interfaces for clinical data exchange with the clinical information system, limiting its direct diagnostic use. In consequence, many institutions now host private cBioPortal instances, allowing researchers to conduct exploratory analyses on their institution-specific data (7).

#### High-Level Technical Overview

The result of the extensions of cBioPortal within UC3, in addition to changes to the cBioPortal user interface, is an extended infrastructure. Since cBioPortal has been split into a frontend and backend part, it now offers a plugin concept that is, however, limited in its functionality. Therefore, the extensions should be coupled as loosely as possible to the codebase to ensure better compatibility with future updates. As a result, updates from the main project frequently may lead to conflicts with custom modifications in the codebase, requiring continual adjustments. Especially challenging are new features such as therapy recommendation, whose implementation affects not only the codebase itself but also the persistence layer. In addition to documenting e g. treatment recommendations through the cBioPortal interface, the resulting data should be stored in FHIR resources to ensure interoperability with a variety of clinical systems (1). Currently, MII-compliant FHIR profiles are being used for this purpose. The result of these extensions is, in addition to changes to the cBioPortal user interface, an expanded infrastructure, see Figure 4 in the manuscript. This has been supplemented with a FHIR server and the middleware Fhirspark and provides the link between cBioPortal and the FHIR server. Fhirspark provides a REST API for this purpose, which allows MTB data in JSON format to be received, enriched with semantic information and transferred to MII-FHIR profiles. This interface is regularly updated to follow the increasingly standardized data model (1,8).

#### Data Handling and Management

CBioPortal is a versatile platform, adeptly supporting various functions related to the exploration and analysis of cancer genomics data. To ensure the accuracy and timely updating of clinical annotations, cBioPortal leverages databases like OncoKB, CIViC, and My Cancer Genome. These sources provide updated, validated clinical actionability information, while tools like the OncoKB annotator help maintain data accuracy through manual curation (1).^[[6]](#footnote-6)^

When discussing the continuous monitoring and reporting of genomic variant changes over time, cBioPortal offers partial support. Users can manually enter treatment start and end dates, enabling tracking of changes and documentation of progress by adding or editing therapy recommendations within the MTB tab. When available, data displays mutations from various samples, allowing analysis of treatment responses by comparing genomic data over time, but this necessitates additional configurations or integrations. Although the platform offers robust visualization capabilities for tracking genomic changes, integrating treatment progress requires further customization (9,10).

While cBioPortal does not independently monitor or validate genomic data quality, it functions as a visualization platform where pre-validation through bioinformatics pipelines ensures data integrity. Uploaded data, in standardized formats like Mutation Annotation Format (MAF), undergoes stringent version control and regular updates managed by institutions. The standardization of the format is not related to the institutions and quality checks/validations are a component of the pipelines used (anyway) (11).

However, the portal includes extensive genomic data, clinical information, and annotations related to targeted therapies and their associated mutations. This can indirectly highlight opportunities for off-label drug use when a mutation or genomic alteration has been shown to respond to a particular therapy. How are germline mutations addressed and their impact on clinical decision-making largely depends on the specific implementation and use within an institution (6). In cBioPortal, though, off-label drug use based on genomic findings isn't directly recorded as an explicit field. The integration of tools like CIViC in cBioPortal help to provide curated data on potential drug responses for specific mutations. Therefore, documenting off-label drug is possible through custom extensions. Users can annotate genomic findings, and integration with databases may enhance this functionality further. Designed to accommodate multiple cancer types and their specific genomic profiles, cBioPortal supports comprehensive dataset integration and customizable data input, utilizing annotations from external databases to facilitate cross-cancer analyses (6,12). While adding a system flowchart could clarify workflows, cBioPortal’s existing structure renders it optional. A flowchart might clarify processes, aid in troubleshooting, and serve as a training tool, yet site-specific workflow variations could limit its universal applicability. Renner et al. suggest that a flowchart's utility heavily depends on individual site setups (1).

#### Data Processing

cBioPortal is an adaptable, platform for exploring cancer genomics, but many capabilities depend on institutional customization and processes and guidelines. Handling variants of unknown significance (VUS) depends on its specific configuration and use within an institution or research setting. Users can view and annotate VUS, but interpretation and clinical decisions typically require expert analysis beyond the portal. The handling of VUS often relies on custom workflows and integrations tailored by the organization using cBioPortal (13). It primarily focuses on somatic mutations related to cancer genomics. However, it can be used to visualize and analyze both somatic and germline mutations when integrated with appropriate data sets and workflows. CBioPortal supports reporting copy number alterations (CNA) and structural variants (SV) using standardized formats, including: 1. MAF: For somatic mutations, including CNAs. 2. Segmented Data Format: Commonly used for CNAs. 3. Variant Call Format (VCF): Primarily for SNPs and small indels, but adaptable for certain large SVs. These formats ensure consistent integration and interpretation of genomic data from various studies. cBioPortal can also be customized to meet specific institutional data requirements.

Data consistency across sequencing platforms is ensured by pre-upload standardization and validation processes. While cBioPortal itself doesn’t allow direct annotations by clinicians for decision-making, extensions can facilitate therapy recommendation documentation, technically, it is possible via (local) therapy recommendations, i.e. our extension, these have not yet been published (1,4). Furthermore, cBioPortal itself does not natively include alert functions to notify healthcare providers of significant genomic findings or changes in patient status. However, such functionality could be developed and integrated into the platform with customization. cBioPortal itself does not inherently provide a mechanism for documenting and resolving discrepancies between recommendations from different tumor boards. The tool is primarily designed for MTBs and is currently limited to local MTBs. Data from other organ boards is included only as timeline data, so there is no need for prioritization. Complex questions still need to be addressed for a collaborative approach across multiple MTBs (9).^[[7]](#footnote-7)^

#### Processes and Integration

cBioPortal’s potential to integrate with EHRs is flexible but requires custom development as it does not natively support seamless EHR integration. Institutions can achieve this integration through extensions like Fhirsparks, which facilitate interoperability. Ensuring patient genomic data privacy when using cBioPortal involves institutional responsibility to configure appropriate measures. They are local (on-premises) installations that are operated within the clinic network of the respective location. The data remains there and is not sent or shared. Strategies such as data anonymization and role-based access controls are essential, with tools like Keycloak providing approval; however, Keycloak offers limited protection outside secure networks. Adherence to data protection regulations like GDPR is contingent upon careful implementation by the hosting institution (1).

Patient consent for genomic testing and data usage must be handled by the institution, as cBioPortal does not store this information. Institutions should establish protocols and workflows to secure informed consent prior to processing genomic data, ensuring compliance with legal standards. Integrating consent management systems can help track and document consents in alignment with data usage policies. Additionally, anonymizing data before upload is essential to protect patient identity and adhere to consent agreements. By implementing these strategies, institutions can effectively use cBioPortal while ensuring compliance with privacy regulations and ethical standards.

cBioPortal provides a flexible and robust platform for exploring and visualizing cancer genomic data, which can indirectly support clinical decision-making in personalized medicine by offering insights into a patient’s genomic profile. However, cBioPortal does not natively include comprehensive clinical decision support tools out-of-the-box (14).

1. Renner C, Reimer N, Christoph J, Busch H, Metzger P, Boerries M, et al. Extending cBioPortal for Therapy Recommendation Documentation in Molecular Tumor Boards: Development and Usability Study. JMIR Med Inform. 2023 Dec 11;11:e50017.

2. Unberath P, Knell C, Prokosch HU, Christoph J. Developing New Analysis Functions for a Translational Research Platform: Extending the cBioPortal for Cancer Genomics. Stud Health Technol Inform. 2019;258:46–50.

3. Unberath P, Mahlmeister L, Reimer N, Busch H, Boerries M, Christoph J. Searching of Clinical Trials Made Easier in cBioPortal Using Patients’ Genetic and Clinical Profiles. Appl Clin Inform. 2022 Mar;13(02):363–9.

4. Reimer N, Unberath P, Busch H, Börries M, Metzger P, Ustjanzew A, et al. Challenges and Experiences Extending the cBioPortal for Cancer Genomics to a Molecular Tumor Board Platform. In: Delgado J, Benis A, De Toledo P, Gallos P, Giacomini M, Martínez-García A, et al., editors. Studies in Health Technology and Informatics [Internet]. IOS Press; 2021 [cited 2024 Feb 27]. Available from: https://ebooks.iospress.nl/doi/10.3233/SHTI210833

5. Ma W, Sartipi K, Sharghigoorabi H, Koff D, Bak P. OpenID Connect as a security service in cloud-based medical imaging systems. J Med Imaging (Bellingham). 2016 Apr;3(2):026501.

6. De Bruijn I, Kundra R, Mastrogiacomo B, Tran TN, Sikina L, Mazor T, et al. Analysis and Visualization of Longitudinal Genomic and Clinical Data from the AACR Project GENIE Biopharma Collaborative in cBioPortal. Cancer Research. 2023 Dec 1;83(23):3861–7.

7. Wu P, Heins ZJ, Muller JT, Katsnelson L, De Bruijn I, Abeshouse AA, et al. Integration and Analysis of CPTAC Proteomics Data in the Context of Cancer Genomics in the cBioPortal. Molecular & Cellular Proteomics. 2019 Sep;18(9):1893–8.

8. Reimer N, Unberath P, Busch H, Ingenerf J. FhirSpark – Implementing a Mediation Layer to Bring FHIR to the cBioPortal for Cancer Genomics. In: Mantas J, Stoicu-Tivadar L, Chronaki C, Hasman A, Weber P, Gallos P, et al., editors. Studies in Health Technology and Informatics [Internet]. IOS Press; 2021 [cited 2024 Oct 28]. Available from: https://ebooks.iospress.nl/doi/10.3233/SHTI210169

9. Buechner P, Hinderer M, Unberath P, Metzger P, Boeker M, Acker T, et al. Requirements Analysis and Specification for a Molecular Tumor Board Platform Based on cBioPortal. Diagnostics. 2020 Feb 10;10(2):93.

10. Ustjanzew A, Desuki A, Ritzel C, Dolezilek AC, Wagner DC, Christoph J, et al. cbpManager: a web application to streamline the integration of clinical and genomic data in cBioPortal to support the Molecular Tumor Board. BMC Med Inform Decis Mak. 2021 Dec;21(1):1–13.

11. Metzger P, Hess ME, Blaumeiser A, Pauli T, Schipperges V, Mertes R, et al. MIRACUM-Pipe: An Adaptable Pipeline for Next-Generation Sequencing Analysis, Reporting, and Visualization for Clinical Decision Making. Cancers. 2023 Jul 1;15(13):3456.

12. Hoefflin R, Lazarou A, Hess ME, Reiser M, Wehrle J, Metzger P, et al. Transitioning the Molecular Tumor Board from Proof of Concept to Clinical Routine: A German Single-Center Analysis. Cancers. 2021 Mar 8;13(5):1151.

13. Barua SA, Goswami N, Mishra N, Sawant UU, Varma AK. *In Silico* and Structure-Based Assessment of Similar Variants Discovered in Tandem Repeats of BRCT Domains of BRCA1 and BARD1 To Characterize the Folding Pattern. ACS Omega. 2022 Dec 13;7(49):44772–85.

14. Park S, Bekemeier B, Flaxman A, Schultz M. Impact of data visualization on decision-making and its implications for public health practice: a systematic literature review. Informatics for Health and Social Care. 2022 Apr 3;47(2):175–93.

1. https://www.medizininformatik-initiative.de/en/basic-modules-mii-core-data-set [↑](#footnote-ref-1)
2. https://ngm-cancer.com/ [↑](#footnote-ref-2)
3. https://pm4onco.de/ [↑](#footnote-ref-3)
4. https://www.toolpool-gesundheitsforschung.de/produkte/mtb-cbioportal [↑](#footnote-ref-4)
5. https://www.mskcc.org/research-advantage/support/digital-health-projects/oncokb [↑](#footnote-ref-5)
6. <https://faq.oncokb.org/data-curation> [↑](#footnote-ref-6)
7. https://www.cbioportal.org/ [↑](#footnote-ref-7)
